# Genetic differentiation of grain, fodder and pod vegetable type cowpeas (*Vigna unguiculata* L.) identified through single nucleotide polymorphisms from genotyping-by-sequencing

**DOI:** 10.1186/s43897-022-00028-x

**Published:** 2022-03-28

**Authors:** Xingbo Wu, Andrés J. Cortés, Matthew W. Blair

**Affiliations:** 1 Department of Agricultural and Environmental Sciences, Tennessee State University, Nashville, TN 37209 USA; 2 Tropical Research and Education Center, Department of Environmental Horticultural, University of Florida, 18905 SW 280th St, Homestead, FL 33031 USA; 3 Corporación Colombiana de Investigación Agropecuaria AGROSAVIA, C.I. La Selva, Km 7 vía Rionegro – Las Palmas, Rionegro, Colombia; 4 Universidad Nacional de Colombia – Sede Medellín, Facultad de Ciencias Agrarias – Departamento de Ciencias Forestales, Medellín, Colombia

**Keywords:** Genomic landscape of divergence, Relative differentiation, *F*_*ST*_, GBS-derived SNP markers

## Abstract

The species *Vigna unguiculata* L. (Walp), commonly known as cowpea, is a multi-purpose legume that has been selected into three subspecies that are divided into grain, fodder and pod (yardlong bean) types. However, genetic bases for distinctions are not well understood. The purpose of this study was to apply genotyping-by-sequencing (GBS) and current reference genome for *V. unguiculata* to distinguish three subspecies and identify signatures of divergence. The collection of 130 accessions included 128 cultivated from: 1) ssp. *cylindrica*, fodder type; 2) ssp. *sesquipedalis*, pod vegetable type; and 3) ssp. *unguiculata,* grain type. Two wilds genotypes from spp. *dekindtiana* and spp. *pubescens*, were used to anchor phylogeny. A total of 11,083 highly informative single nucleotide polymorphisms (SNPs) were discovered. Wild accessions showed distinct genetic fingerprints and were separated from cultivated subspecies. Principal component analysis showed closer relationship between ssp. *unguiculata* and ssp. *cylindrica* compared to ssp. *sesquipedalis*. Relative differentiation of cultivated subspecies (with Fixation Index, *F*_*ST*_) indicated the existence of discrete signatures of selection. This work clarifies the population structure, phylogeny, and domestication of cultivated cowpeas. Furthermore, significant genetic differences between grain and pod vegetable types can provide valuable information for future breeding in three cowpea groups.

**Supplementary Information:**

The online version contains supplementary material available at 10.1186/s43897-022-00028-x.

## Core

 Analyzing 130 accessions of fodder, grain, vegetable and wild cowpeas with GBS technology, we found 11,083 SNPs. Wild accessions were distinct from the three cultivated types, while there was a close genetic relationship between fodder and grain subspecies with vegetable cowpeas derived from a subset of these. This work clarifies the population structure footprints of the three cultivated cowpea subspecies and provides valuable information for future breeding improvements targeting genetic differences between grain and vegetable cowpeas.

## Introduction

Cowpea (*Vigna unguiculata* L. Walp.) is a widely cultivated legume species of the tribe *Phaseoleae* with a small diploid (2*n* = 2*x* = 22) genome. The species is economically important as a multi-purpose crop useful as fresh shelled horticultural pea or dry grain (pulse), fodder (feed) and pod vegetable (yardlong bean) crops in different parts of the world (Ehlers and Hall, 1997). This distinction parallels the three main subspecies defined for *V. unguiculata*: 1) the vegetable genotypes used for their long pods are from the ssp. *sesquipedalis*, 2) the pulse genotypes used for their dry grain or immature seed are from the ssp. *unguiculata*, and 3) the small seeded fodder type are mainly from the ssp. *cylindrica,* although some of the ssp. *unguiculata* are used for feeding animals such as goats (Lush et al., 1980). The pulse crop is grown across many countries around the world but is an everyday staple in West Africa, especially in Senegal, Nigeria, Niger, Mali, Ghana, the Gambia and Burkina Faso (Hall et al., 2003). More minor production of field cowpeas for fresh seeds or dry grain, locally consumed or for export, is reported in Brazil, the Caribbean, India and Pakistan, the Middle East, Venezuela and the United States (Singh et al., 2002). Among the states that produce the crop on a significant scale are Alabama, Arkansas, California, Florida, Georgia, Mississippi, Missouri, North Carolina, Oklahoma, South Carolina, Tennessee and Texas, in many cases overlapping with areas of African American populations.

Meanwhile, the pod vegetable crop is common and economically very important in East Asia, especially in China, and in South / Southeast Asian countries (Bangladesh, Cambodia, India, Laos, Nepal, Thailand, Vietnam). The yardlong bean vegetable cowpea type, often is called asparagus bean as well, is unique because of its long pods (up to 100 cm in length) and climbing growth habit, which is very distinct from the shorter pods (8 to 16 cm) and bush growth habit of the dry grain type (Xu et al., 2017). The yardlong bean can be produced year-round in greenhouses and is high yielding as the tender pods can be harvested up to four times in a short season (Kongjaimun et al., 2012). The viny yardlong beans are grown on trellises in Asia and sometimes in sub-tropical parts of the United States, but their production is less frequent in Africa or Europe (Sprent et al., 2010). Therefore, the species *Vigna unguiculata* is found as far north as the central region of China and midwestern states of the USA, although much of the small export market is in desert production under irrigation in California and Peru. Around the world the majority of cowpea production is for local consumption either as subsistence grain or fresh vegetable.

The centers of origin and diversity of cultivated cowpea are known to be in Africa, but they are diffuse, with wild relatives found in both the western and southern parts of the continent (Fatokun et al., 2018). The spp. *unguiculata* group was thought to have been domesticated in the watershed of the Niger river in West Africa, and then spread throughout much of Sub-Saharan Africa over four thousand years ago (Rawal, 1975). Most likely, cowpeas went to South Asia at some point from East Africa when traders started linking the two continents (Lush and Evans, 1981). Starting four hundred years ago, the cowpea spread into Northeast Brazil, the Caribbean and the Southeastern United States with the beginning of the African diaspora (Huynh et al., 2013).

The vegetable (long pod) cowpeas of the *sesquipedalis* group are believed to have been derived from the non-vegetable (short pod) cowpeas of the spp. *unguiculata* group somewhere in South/Southeast Asia, possibly in India (Lush et al., 1980). Even though the genetic bases of spp. *unguiculata* and spp. *sesquipedalis* are very similar, the extent of genome diversification between the two subspecies is not well understood, and many seed types are seen in both subspecies varying in color and patterns, however with the vegetable type seed longer to fit in the long pods (Xu et al., 2011). On the other hand, the spp. *cylindrica* is a subgroup notable for round dark-colored or gray seeds, and has been used as a fodder crop. Unlike spp. *unguiculata* does not have the oblong, light color seeds characteristic of the more common genotypes grown for the other two purposes, grain or vegetable (Lush et al., 1980; Sahay and Shukla, 2020). The relationship of this subspecies to the other two is poorly understood.

Studies aimed at understanding the genetic diversity of cowpea subspecies have been reported using several types of molecular markers, including isozymes (Reis and Frederico, 2000), random amplified polymorphic DNA (RAPD) (Malviya et al., 2012), amplified fragment length polymorphism (RFLP) (Fang et al., 2007), and simple sequence repeats (SSRs) (Olasupo et al., 2018). Though genetic variations were detected within this species, only partial conclusions could be drawn on the origin of different cowpeas due the limited number of markers originated from certain genomic regions. GoldenGate assays of 1,536 single nucleotide polymorphism (SNP) makers identified two cowpea gene pools with 422 cowpea accessions collected from 56 countries (Huynh et al., 2013), but only grain cowpea (*V. u.* subsp. *unguiculata*) and its wild relative (*V. u.* subsp. *dekindtiana*) were included. Recently, a more robust study with 51,128 SNPs has analyzed a core collection at University of California Riverside but only with cultivated cowpea accessions (Munoz-Amatriain et al., 2021).

Another method for evaluating large numbers of SNPS is a procedure named genotyping-by-sequencing (GBS), introduced about a decade ago as a more reliable tool in plant genetics compared to previous markers systems (Elshire et al., 2011). This method is based on restriction enzymes, which enable the creation of reduced genome complexity libraries used in multiplexing next generation sequencing (NGS). With the assistance of appropriate bioinformatics filtering tools and genome information, GBS has achieved genome level SNP discovery and diversity evaluation in large germplasm collections at low cost (Poland and Rife, 2012). It has been widely used in multiple crops for genetic diversity, linkage mapping, genomic prediction (GP), genome wide association studies (GWAS) (He et al., 2014) and genome wide selection scans (GWSS) (Cortés et al., 2018). GBS has been adopted for genetic studies to discover SNPs associated with salt tolerance in cowpea (Ravelombola et al., 2018). Furthermore, GBS was also used to generate a set of cowpea core collection comprised of 298 lines to encompass cowpea diversity at the International Institute of Tropical Agriculture (IITA) (Fatokun et al., 2018).

Despite advances, neither of the previous GBS studies in cowpea could reveal a complete SNP information due to the absence of cowpea genome information at that time. In this study, we used GBS analysis to understand the differences between the three main spp. of *V. unguiculata* representing grain cowpea, fodder cowpeas and vegetable yardlong bean. To determine variability across the genome we used the approach of aligning GBS fragments with a full cowpea reference genome sequence that has recently become available (Lonardi et al., 2019). We then used the detected SNPs as a comprehensive way of evaluating genotypic diversity across a broad collection of the three types of cowpeas. The use of a reference genome was found to improve the precision of the GBS technique for discovery SNPs as well as structural variants such as insertion/deletion events. The boundaries between the spp. were clearly delineated by our germplasm analysis. Knowledge of the extent and pattern of genetic diversity and past phylogeographic selection provides plant breeders with an opportunity to develop new varieties with desirable traits.

## Results

### SNP discovery and distribution

In total, 63,947 raw polymorphisms were identified through alignment of the GBS tags with the newest cowpea reference genome available for *V. unguiculata* made for the genotype IT97K-499-35. Among them, many polymorphisms (82.7%) were indels, had missing data or low allele frequency, and were removed from the dataset. This level of depuration is typical from GBS and NGS technologies and aim retaining high quality markers. After this, 11,083 high quality SNPs were retained for further analyses. The high quality SNP loci were distributed across the cowpea genome with an average of one SNP every 21.3 Mbp of genomic sequence. Furthermore, they were spread out across the entire set of chromosomes (Table 1), although they varied in average density per chromosome from one SNP every 15.4 Mbp on Vu09 to one SNP every 22.8 Mbp for Vu10. Overall, a total of 8,795 (79.7%) high quality SNPs were discovered on contigs mapped directly to the genome and corresponding to known positions on the 11 chromosomes of the cowpea genome. Correspondingly, a total of 2,288 (20.6%) high quality SNPs were located on unmapped sequence contigs from the reference genome. Therefore, the effective average SNP density on mapped contigs was 18.66 ± 2.35 SNP/Mbp across the mapped cowpea genome. Distribution of the SNPs was even across each chromosome, but clustered near the sub-telomeric regions (Fig. S1). The full SNP information was deposited with the BioProject accession number PRJNA664609 in the SRA database.

**Table 1:** Number of high-quality single nucleotide polymorphism (SNP) loci found by genotyping-by-sequencing

Chromosome	Number of SNPs	Chromosome Size (Mbp)	SNP Density (SNP/Mbp)
Vu01	735	42.13	17.45
Vu02	568	33.91	16.75
Vu03	1,142	65.29	17.49
Vu04	750	42.73	17.55
Vu05	878	48.75	18.01
Vu06	763	34.46	22.14
Vu07	754	40.88	18.44
Vu08	695	38.36	18.12
Vu09	678	43.93	15.43
Vu10	941	41.33	22.77
Vu11	891	41.68	21.38
Unmapped	2,288	45.98	49.76
**Total**	11,083	519.43	21.34

Statistics are shown across the eleven chromosomes and unmapped contigs of the *Vigna unguiculata* (IT97K-499-35) cowpea reference genome, and their density based on reported chromosome sizes in mega base-pairs (Mbp).

### Principal component analysis of three types of cowpea accessions

**Fig. 1:**
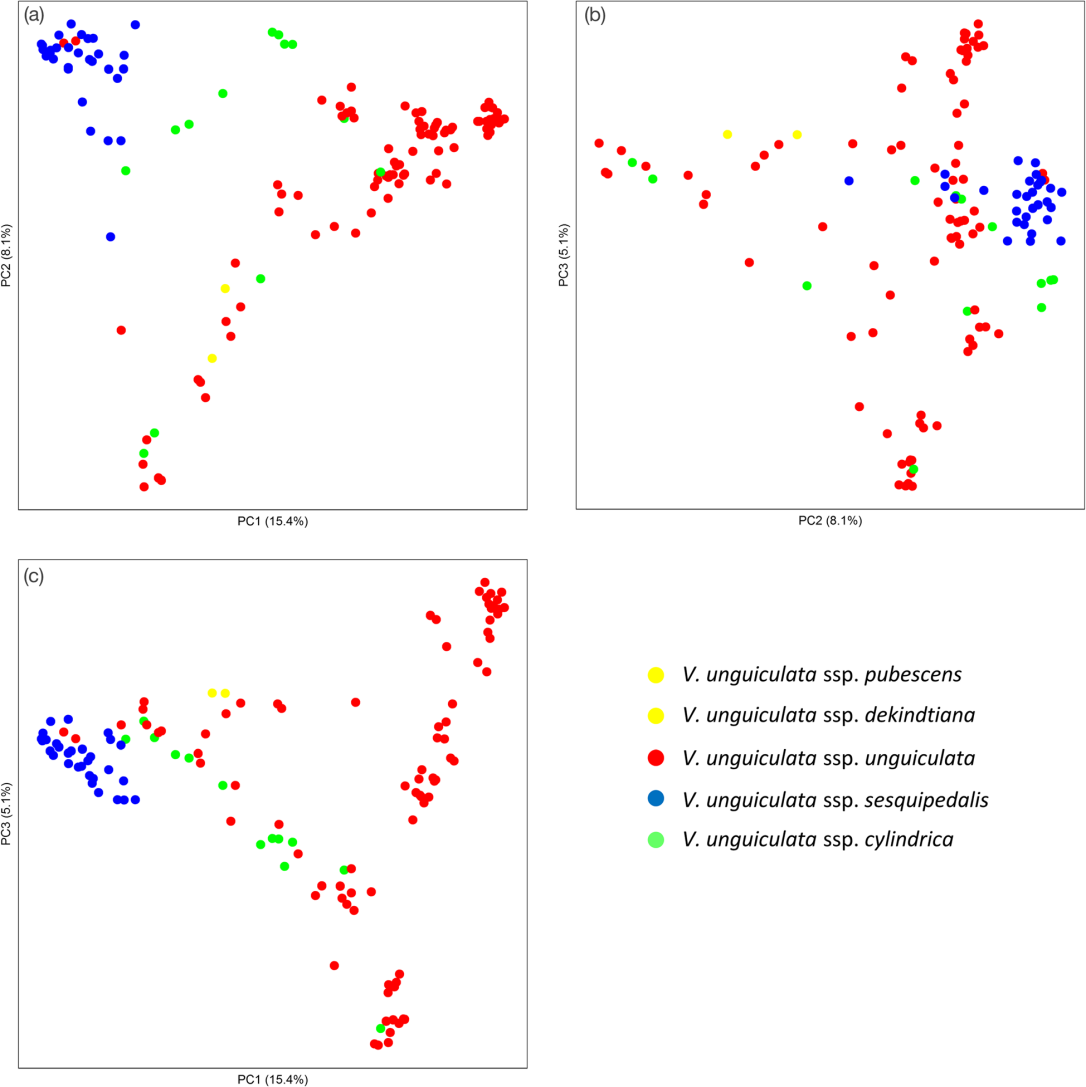
Visualization of the genetic relationships among three domesticated cowpea subspecies by Principal Component Analyses

The genetic principal component analysis (PCA) identified differentiation among three subspecies and their wild relatives, with the main difference being between yardlong beans (blue dots) compared to grain type cowpeas (red dots), forage cowpeas (green dots) being intermediate (Fig. 1). The three axes of the PCA explained 15.4% (PC1), 8.1% (PC2) and 5.1% (PC3) of total variation, respectively. Two wild relatives, represented by two shades of yellow dots, were identified as intermediates from the comparisons between each of the two Axes. Comparison between PC1 and PC2 differentiated spp. *sesquipedalis* (yardlong bean) accessions as one cluster. This group was different from spp. *unguicalata* and spp. *cylindrica* (grain and fodder cowpea) accessions, respectively. Grain cowpeas could be separated into two sub-clusters by mixing with fodder cowpea accessions. Two grain cowpea accessions were also clustered with yardlong bean, and were the same ones identified in the phylogenetic study as being related to spp. *sesquipedalis*. The PCA resulted in groups that broadly agreed with the subspecies identification and classification types of the cowpeas, namely as vegetable (spp. *sesquipedalis*), fodder (spp. *cylindrica*) or grain (spp. *unguiculata*) genotypes.

### Population structure of cowpea accessions

**Fig. 2:**
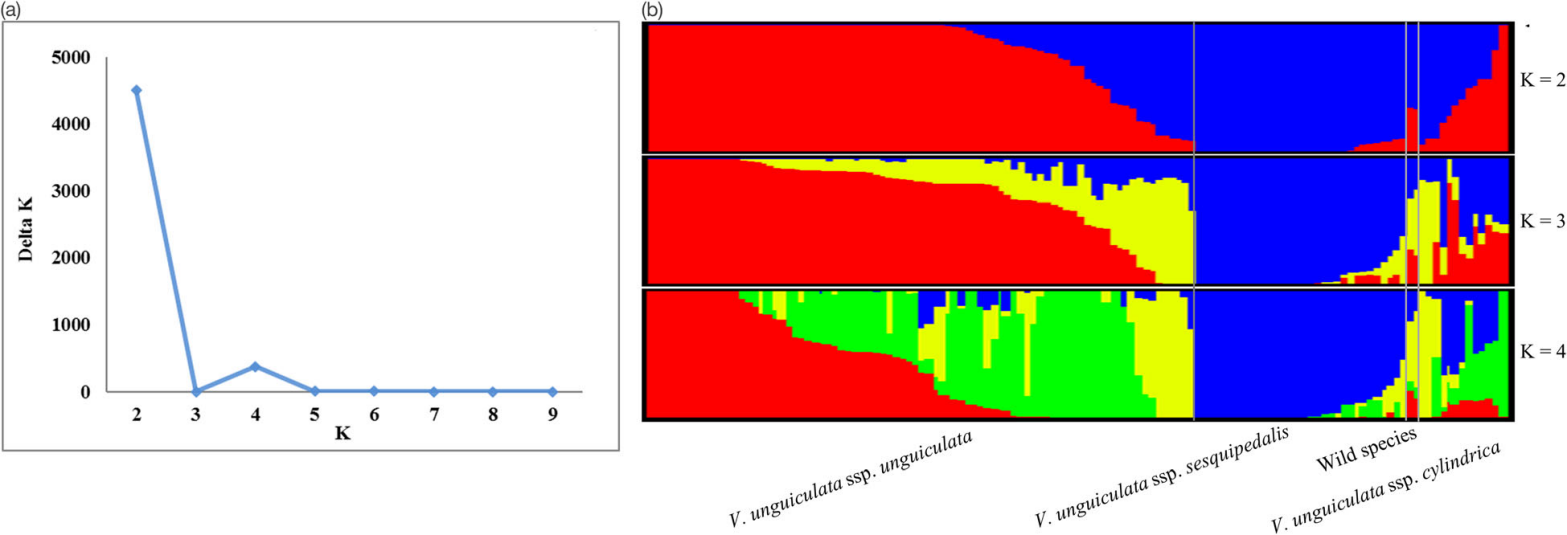
Population structure analysis for 130 cowpea accessions. **A** Plot of the *ΔK* value with the number of subpopulations (*K*) from 2 to 9 based on the Evanno test. **B** Distruct plot with each accession represented by a vertical line, and cluster assignments indicated by color

The population structure of three cultivated cowpea subspecies and their wild relatives was also evaluated by unsupervised Bayesian clustering analysis assuming an admixture model. Valid subpopulation numbers were identified by the Evanno test. An initial *ΔK* peak was found for *K* = 2 (Fig. 2A) which divided cowpea accessions into two genetic groups, followed by a secondary peak at *K* = 4, indicating four sub-groups. This varied from what presented in the phylogenetic analysis; and therefore, separation of subgroups at each intermediate *K* value are presented.

At *K* = 2 (Fig. 2B), 95 accessions (73.08%) were assigned to two groups with a membership likelihood > 0.85 (Table S1). The grouping was mainly based on the taxonomy classification and growth type (bush or climbing type). The ssp. *unguiculata* collection (grain type) was divided into two groups, with 54 accessions clustered into the first group and eight US breeding lines into the second group. All ssp. *sesquipedalis* accessions (vegetable type) were categorized into the second group. Out of 13 ssp. *cylindrica* accessions (fodder type), one accession from Nigeria showed high similarity with ssp. *unguiculata* accessions and cluster with the first group, while two accessions from China and Guatemala were clustered with ssp. *sesquipedalis* accessions as the second group. Meanwhile, the ssp. *cylindrica* accessions exhibited rampant introgression and did not differentiate into a single group at *K* = 2. Meanwhile, at *K* = 3, the main three groups of interest: agronomic grain type, *V. unguiculata ssp. unguiculata*, fodder types (spp. *cylindrica*) and vegetable pod type (ssp. *sesquipedelis*), showed different amounts of genomic admixture; showing as with *K* = 2 that yardlong beans are derived from grain cowpeas not from fodder types, although fodder types could be a source of genes for both of grain and vegetable cowpeas.

At *K* = 4, the ssp. *unguiculata* collection was divided into four subgroups. All accessions from Niger, along with 3 from Nigeria, 3 from Cameroon, 2 from Senegal, one from Burkina Faso and an IITA breeding line (“Dan lia”) formed one group. The second group comprised of 9 accessions from Nigeria, 3 from Ghana, 2 US breeding lines (“524B” and “CB27”) and one each from Benin, Botswana, Burkina Faso and Côte d’Ivoire, which were predominantly of African origin. The third group has five accessions with one each from Egypt, Iran, Mali, Nicaragua and Tanzania; an unusual set of countries that may have received germplasm from a single source program. Two accessions that were clustered separately from other ssp. *unguiculata* collections (*K* = 2) remained independently as the fourth group, along with all ssp. *sesquipedalis* collection. In ssp. *cylindrica* collection, one accession from Nigeria and two accessions from Honduras and Turkey were clustered into the second and third group, while other accessions showed mixture of all groups. No valid group was clustered at *K* = 3 with minimum membership likelihood. Two wild accessions remained admixture at both *K*s.

### Phylogenetic analysis

**Fig. 3:**
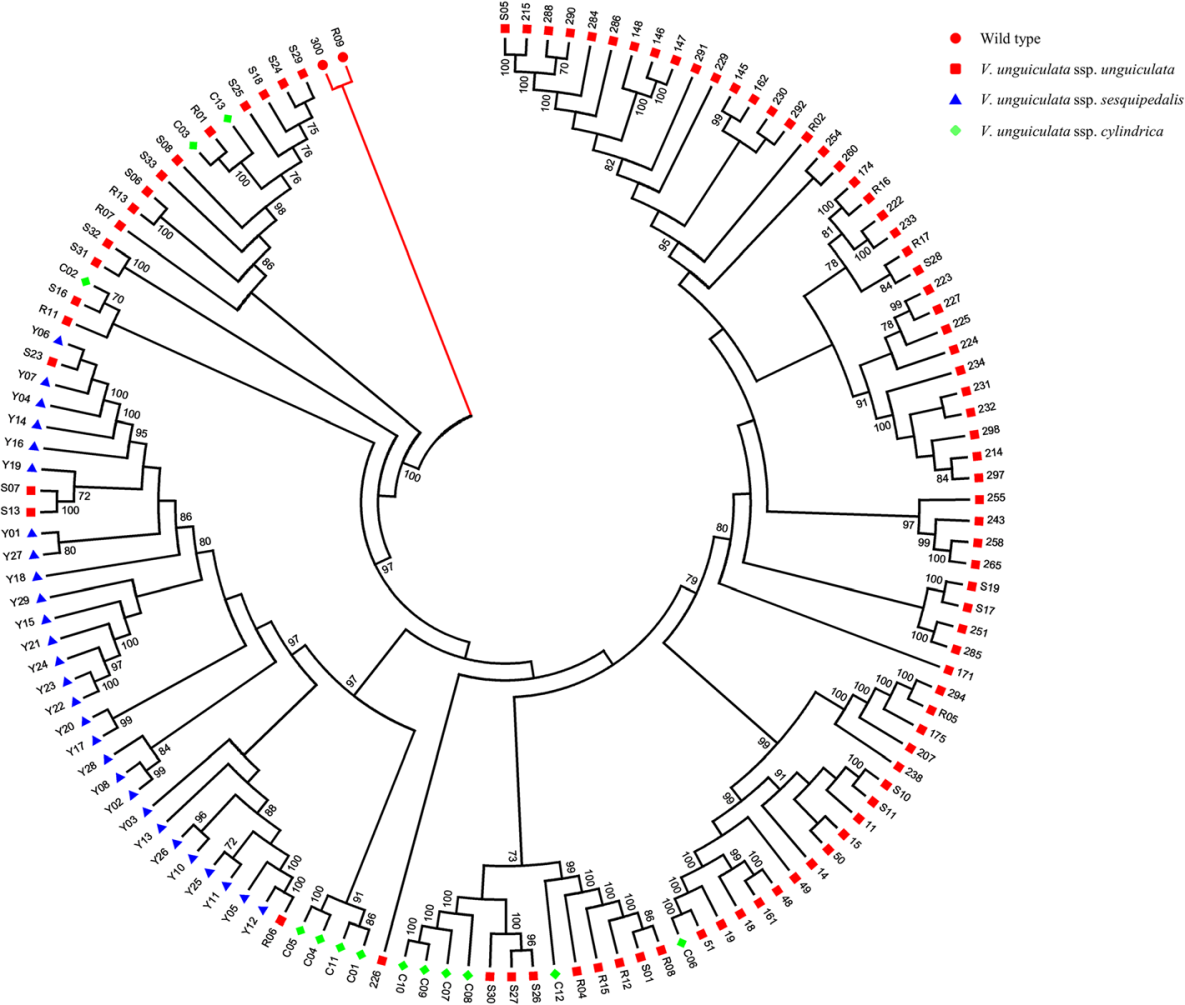
Rooted phylogenetic tree of 130 accessions from three cowpea subspecies and two wild relative species. Bootstrap values > 50% are marked above nodes

A maximum likelihood phylogeny (Fig. 3) was generated for all the genotypes using the set of 11,083 high quality SNP markers and 500 bootstrap replications to provide branch node probability values. The analysis considered both mapped and unmapped SNP markers with the assumption that unmapped contigs and the SNPs they contain will be joined to mapped contigs and chromosomal pseudomolecules in future builds of the reference genome. Some ssp. *unguiculata* were grouped with ssp. *sesquipedalis* likely due to incomplete lineage sorting. Alternatively, this may reflect the introgression detected as part of the unsupervised Bayesian clustering approach used. A final biological explanation could be that a few grain types gave rise to the vegetable types, and the accessions grouping with the spp. *sesquipedelis* are related to that ancestral founder group for the horticultural type, what is now called the yardlong bean. Geographic analysis might show that East African cowpea germplasm made it to South and Southeast Asia, where yardlong bean was developed from previously domesticated grain cowpea types, but having especially long pods produced on climbing trellised type plants rather than shorter pods of grain types, which can be sprawling or compact in plant type.

Three major clusters were identified in the maximum likelihood phylogeny apart from the outgroup. All the vegetable cowpea (ssp. *sesquipedalis*) accessions, along with two grain cowpea accessions, were separated and clustered into a group from South Asia. The grain cowpea (ssp. *unguiculata*) and fodder cowpea (ssp. *cylindrica*) accessions were clustered into two separate groups. Accessions from Southern and Central Africa formed into one group, and the remaining accessions encompassed genotypes with origins as diverse as Africa, the Americas and Asia.

### Genetic diversity and genomic landscape of divergence of three subspecies

When the relative divergence (*F*_*ST*_) score (Fig. S2) was compared between three cowpea subspecies (Fig. S3), the highest *F*_*ST*_ values were observed between grain (ssp. *unguiculata*) and yardlong cowpea (ssp. *sesquipedalis*), while the lowest *F*_*ST*_ was detected between grain (ssp. *unguiculata*) and fodder cowpea (ssp. *cylindrica*).

Though distinct *F*_*ST*_ patterns were observed in three comparisons, higher divergence tended towards the extremes of the chromosomes of each comparison, where linkage disequilibrium (LD) also tended to decay. Given that the small chromosomes of *V. unguiculata* are like those of common bean (*Phaseolus vulgaris* L.), similar genomic diversity patterns are seen (Cortés and Blair, 2018), with reduced recombination within the center of each chromosome and crossovers pushed to near the chromosome ends, where LD decays. Inter-crossability of all three types of cowpeas indicates they have similar genomes, although the variability in genome structure between subspecies remains to be analyzed. The nucleotide diversity (π) showed a random pattern across the genome overall, as well as for each subspecies specifically (Fig. S4).

Signatures of divergence were identified in the *F*_*ST*_ comparison between three subspecies with a type-I error rate (permissible α value) of 0.001 (Fig. S3). A total of 27 outlier regions for *F*_*ST*_ between ssp. *unguiculata* and ssp. *sesquipedalis* were detected on seven chromosomes, encompassing 270 genes. Meanwhile four outlier regions for *F*_*ST*_ between ssp. *sesquipedalis* and ssp. *cylindrica* were detected in two chromosomes, encompassing 23 genes (Table 2, Table S2). No outlier *F*_*ST*_ regions were detected when comparing grain and fodder cowpea.

**Table 2:** Summary of peaks of relative differentiation (based on the average *F*_*ST*_ score)

Pairwise comparison	Chromosome	Start (bp)	End (bp)	Length (bp)	Mean FST	Flanking region	Genes	Known QTL
**Grain vs. Vegetable**	Vu02	24,602,281	24,602,470	189	0.87	24,552,281–24,652,281	9	
	Vu03	7,433,261	7,433,390	129	0.87	7,383,261–7,483,261	11	
	Vu03	13,546,721	13,547,040	319	0.89	13,496,721–13,596,721	9	
	Vu03	13,619,671	13,619,860	189	0.90	13,569,671–13,669,671	7	
	Vu03	13,785,361	13,785,590	229	0.88	13,735,361–13,835,361	6	
	Vu03	13,793,301	13,793,510	209	0.88	13,743,301–13,843,301	4	
	Vu03	13,795,501	13,795,770	269	0.88	13,745,501–13,845,501	4	
	Vu03	14,389,911	14,390,100	189	0.90	14,339,911–14,439,911	10	
	Vu03	14,571,291	14,571,480	189	0.89	14,521,291–14,621,291	8	
	Vu04	2,898,691	2,898,880	189	0.87	2,848,691–2,948,691	10	
	Vu06	20,197,641	20,197,830	189	0.87	20,147,641–20,247,641	11	
	Vu06	22,810,701	22,810,890	189	0.87	22,760,701–22,860,701	12	
	Vu06	23,226,891	23,227,080	189	0.90	23,176,891–23,276,891	15	
	Vu06	23,349,871	23,350,000	129	0.93	23,299,871–23,399,871	12	
	Vu08	32,138,851	32,139,040	189	0.90	32,088,851–32,188,851	14	
	Vu08	33,036,301	33,036,620	319	0.93	32,986,301–33,086,301	13	
	Vu08	33,073,211	33,073,310	99	0.92	33,023,211–33,123,211	8	
	Vu08	33,103,051	33,103,240	189	0.91	33,053,051–33,153,051	10	
	Vu08	33,264,011	33,264,200	189	0.91	33,214,011–33,314,011	14	
	Vu08	33,278,221	33,278,410	189	0.87	33,228,221–33,328,221	15	
	Vu08	36,035,191	36,035,380	189	0.89	35,985,191–36,085,191	20	Pod length^42^
	Vu10	33,225,011	33,225,200	189	0.93	33,175,011–33,275,011	11	Seed coat pattern^41^
	Vu10	33,226,961	33,227,280	319	0.91	33,176,961–33,276,961	12	Seed coat pattern^41^
	Vu10	36,476,061	36,476,250	189	0.88	36,426,061–36,526,061	9	Seed coat pattern^41^
	Vu10	36,693,191	36,693,400	209	0.93	36,643,191–36,743,191	11	Seed coat pattern^41^
	Vu11	23,872,761	23,872,950	189	0.88	23,822,761–23,922,761	6	Flower scent^42^
	Vu11	26,358,441	26,358,630	189	0.87	26,308,441–26,408,441	5	Flower scent^42^
**Vegetable vs. Fodder**	Vu03	14,561,131	14,561,290	159	0.87	14,511,131–14,611,131	7	
	Vu09	15,507,411	15,507,600	189	0.90	15,457,411–15,557,411	5	Seed coat pattern^42^
	Vu09	16,061,581	16,061,770	189	0.89	16,011,581–16,111,581	4	Seed coat pattern^42^
	Vu09	16,189,361	16,189,550	189	0.88	16,139,361–16,239,361	1	Seed coat pattern^42^

Table footnote: Flanking regions, candidate genes, and known QTLs are shown among different subspecies of cowpea. Columns start, end and length correspond to the peaks of divergence, mapped according to the midpoints of consecutive outlier windows, while the flanking region corresponds to the entire 100 kb flanking window surrounding the peak of divergence. The gene search is carried out in the latter.

Outlier divergent regions were found to overlap with reported QTLs that were related to pod length, flower scent and seed coat pattern in cowpea (Table 2). One significant outlier region for divergence in the comparison of ssp. *unguiculata* and spp. *sesquipedalis* was on Chromosome Vu08, overlapping with a pod length QTL (Lo et al., 2018). This might be expected since vegetable type cowpeas were much longer podded than grain types. This, despite having a similar number of seeds per pod, indicates that vegetable types were derived from an elongation of the ovary/placenta leading to greater pod (fruit) length rather than an increase in number of ovules/seed per pod.

A previously identified seed coat pattern QTL overlapped with three regions under selection on Chromosome Vu09 and four regions under selection on Chromosome Vu10 (Herniter et al., 2019). Why specific seed coat color would be associated with yardlong beans is not known, but in snap beans (*Phaseolus vulgaris* L.) light seed colors are often favored by consumers and the same could be playing a role in cowpea (*Vigna unguiculata*) derived vegetables. The flower scent QTL overlapped with two outlier regions on Chromosome Vu11 (Lo et al., 2020). This latter QTL associated with flower scent might be driver for reproductive isolation among the three types of cowpeas. In contrast, the other QTLs more likely corresponded to *ad* *hoc* co-adaptation as part of contrasting uses and domestication forces.

Overall, the vegetable types were more diverged and highly selected compared to the two other cowpea types. We did not find evidence for gene clusters such as Resistance Gene Analogs controlling the differences between the types of cowpea. However, we did find that two clusters of linked seed coat pattern genes on Vu10 differentiated grain and vegetable types, while one cluster of seed coat pattern genes on Vu09 differentiated fodder and vegetables types.

## Discussion

The main achievement of this study was to compare morphologically defined subspecies within *Vigna unguiculata* for the validity of their genetic distinctiveness based on a large set of genome wide SNP markers from GBS evaluation. Though previous genetic studies have been conducted in cowpea accessions using the GBS approach (Xiong et al., 2016; Fatokun et al., 2018), those studies lacked non-grain cowpea genetic resources, such as vegetable and fodder cowpea accessions, and did not map most of the discovered SNPs. In the current study, a total of 11,083 high quality SNPs were identified based on cowpea genotypes from three subspecies and mapped onto the newly released reference genome. This nearly doubled the number of SNPs compared to the past GBS study showing effectiveness with the reference genome. Moreover, the use of newly released genome sequence resources also provided complete information on each SNP, such as position and flanking sequences. This information adds additional value to the newly discovered SNPs, as these markers can be used to map chromosome regions and even genes that are responsible for important agronomic traits through linkage and QTL studies, which can in turn be converted to breeder-friendly tools to facilitate marker-assisted selection in cowpea.

Another useful aspect of our study was the use of an outgroup, made up of two wild relative cowpea subspecies (ssp. *dekindtiana* and ssp. *pubescens*), to anchor the three cultivated subspecies from *V. unguiculata* in a maximum likelihood phylogenetic analysis. We showed high genetic differentiation between the wild and cultivated cowpea subspecies, and moderate distinctiveness of the vegetable, fodder and grain types from each other. Wild accessions have been important in providing desirable genes to improve cowpea varieties with better resistance to biotic and abiotic stresses in several breeding programs (Andargie et al., 2011). However, crosses between the wild and cultivated have been infrequent, and even more so crosses between the vegetable and non-vegetable types, which have rarely been made.

The analyses of the vegetable types from the ssp. *sesquipedalis* group, showed its distinct genetic composition in our study. The yardlong bean accessions from this subspecies were separated from other cowpea subspecies accessions in the maximum likelihood clustering, which agreed with PCA results. In the population structure analysis, this subspecies was always distinct, even when assuming two, three or four subpopulations. Some accessions from ssp. *unguiculata* and spp. *cylindrica* shared some genome similarity in the various groupings. Overall, the clustering and grouping phenomena observed here indicated the unique genetic background of the vegetable subspecies in comparison to other cultivated cowpea, and its distinctiveness from fodder or grain types. This may indicate that the yardlong bean was derived from a different source than the other two types, perhaps from a different region such as East Africa, compared to the grain and fodder types more commonly used as a staple pulse crop in West Africa.

Indeed, yardlong bean, which now constitutes the ssp. *sesquipedalis*, is thought to be domesticated from an unidentified group of cultivated cowpea (*V. unguiculata ssp. unguiculata*) at an uncertain time and from an unknown source or cowpea germplasm from Africa introduced to South Asia (Ng and Marechal, 1985). This subspecies contained distinct morphological traits when compared to the other cultivated cowpea subspecies, with one of the them being the extremely long pods up to one meter in length, making it known as the yardlong bean in Asian countries (Verdcourt, 1970). The other unique traits that differentiate the yardlong bean is the complete loss of pod shattering, which is one of the primary traits for the domesticated wild legumes (soybean, common bean, azuki bean, rice bean and mung bean), as described in (Parker et al., 2021). This subspecies may reflect the intense breeding pressure between their introduction from Africa to Asia, both in terms of environmental adaptation and man-made selection. In the study, we included all available vegetable cowpea (ssp. *sesquipedalis*) and forage cowpea (ssp. *cylindrica*) accessions that were available in the core collection of the USDA germplasm bank. However, greater sampling would be useful in future studies to look at diversity within each subspecies and each cowpea type, as well as to better reveal signatures of genomic selection.

In Africa, cowpea grows in the hot and drought-susceptible environments, where abiotic stresses and insect pests have been the main limitations. This contrasts with the humid and high rainfall environments of monsoonal Asia where yardlong bean is grown and diseases such as powdery mildew have been the primary concerns. As the dry grain is the main type of cowpea in Africa, high yield, non-shattering pods and predictable maturity were the most preferred traits, along with nutritious and fast cooking seed for this type. In many cases the dry grain types in Africa are dual purpose with the vines used for goat feed, so vinyness and biomass were also preferred, balanced with the number and timing of seeds produced. In comparison, the main traits selected for vegetable demands in Asia were tenderness of the immature pods, high pod production, pod length and slow elongated seed development fitting into the elongated pods.

The ssp. *unguiculata* collection showed a diverse distribution in both maximum likelihood phylogeny and population structure analyses. A total of four groups were observed in this subspecies. Overall, accessions from Nigeria and Niger were assigned into group I and II, which were also observed in two other studies where different marker systems and germplasm sets were used (Isemura et al., 2010; Fatokun et al., 2018). The division of Nigeria accessions in two groups was not surprising as evidences had shown the sub-region of west and central Africa is diversity center of cultivated cowpea (Coulibaly et al., 2002; Chen et al., 2017). Current U.S. cowpea breeding lines were divided into two groups, where seven west African countries were assigned into Group II and five accessions from different geographic regions (Iran, Mali, Nicaragua, Tanzania and Egypt) were assigned into Group III. It has been suggested that cowpea came to the U. S through the slavery from West Africa, and thus parental lines for cowpea breeding were of West African origin in the USA, especially in the Southeastern states (Whit, 2007).

The observation of different geographic sources of the breeding lines other than west Africa region justified the wide genetic representation of U.S. accessions. Since California has become the main export quality cowpea producer (Huynh et al., 2013), we also included 36 cowpea accessions that were extensively used for cowpea genetic analyses in the past few years from University of California at Riverside and at Davis. The majority of cowpeas in IITA’s international germplasm bank are undefined for subspecies or are defined as grain types from a few major cowpea pulse producing countries such as Nigeria and Ghana. Since, we did not want to over-sample from the grain type group more than was necessary, we made sure to include 24 cowpea references accessions representing the diversity described in Huynh et al. (2013). It should be noted that many accessions in international or even U.S. collections, outside the core collection, have mixed seed colors and represent marketplace populations or landraces, not pure varieties. Therefore, for both grain and vegetable types we selected single seed type purifications from the USDA collection to represent most diversity at either academic or government institutions in the United States.

The population structure analysis identified optimal group number (*K*) being 2. Interesting admixture at *K* = 3 and a secondary ideal number at *K* = 4 suggests subdivisions events (Fig. 2) in the three cultivated subspecies representing grain, fodder and vegetable cowpeas. The spp. *sesquipedalis* yardlong beans remained as one group regardless of the assigned *K* values from 2 to 4. In spp. *unguiculata* dry grain cowpea, the first group (*K* = 2) further divided into two subgroups mainly based on the geographic origin, represented by accessions from Niger and Nigeria, compared to those from other African or tropical countries in Asia and the Americas. Nigeria is a main target country for breeding by the IITA program and could represent this emphasis as dry grain breeding lines were mainly from that program based in Kano (Boukar et al., 2019).

Interestingly, two cowpea breeding lines (“M101” and “TVu-6644”), though characterized as ssp. *unguiculata*, were grouped with ssp. *sesquipedalis*. Morphologically, these two genotypes showed climbing growth habit, similar to the ecotype of vegetable cowpea, indicating they may share a strong genetic background with ssp. *sesquipedalis*. The maximum likelihood phylogeny presented a close genetic relationship between ssp. *cylindrica* and ssp. *sesquipedalis* (Fig. 3), which was also observed in another study (Gillaspie et al., 2005). These two subspecies were both cultivated in Asia but for different agronomic purposes (Lush et al., 1980; Lush and Evans, 1981). In Asia, the ssp. *cylindrica* is used mainly as fodder to feed animal or cover crop to suppress weeds, where the primary traits are high biomass and quality for animal nutrition (Yadav et al., 2018).

The higher divergence towards the extremes of the chromosomes, where LD decays, along with the random distribution of nucleotide diversity pattern, indicate genomic footprints of breeding and/or adaptation pressures among all three subspecies of cowpea. This is because evolutionary theory predicts that when high divergence and low recombination do not match, divergence is mostly driven by selective/adaptive forces, rather than genetic drift or genomic constrains (Ellegren and Wolf, 2017).

On the other hand, when divergence and suppressed recombination coincide, divergence is likely due to the enhancement of lineage sorting relative to background levels (Wolf and Ellegren, 2017) by a reduction in the effective population size (*Ne*), which ultimately expands throughout the genomic vicinity via genetic hitchhiking (Ma et al., 2018) and linked selection (Desalegne et al., 2016). The latter phenomenon is what has been observed in common (Cortés and Blair, 2018) and lima bean, in which higher divergence matches low-recombining regions (Cortés et al., 2018). Low effective population size and drift can also cause deviations of LD values. Areas of divergence can be searched for when exploring the genomic landscape for the footprints of adaptive trait selection, something we were most interested in when comparing the different types of cowpeas.

Among the regions of divergence between grain and vegetable type cowpea, two regions were related to previously identified QTL for pod length and flower scent on Vu08 and Vu11, respectively. Nine genes were found on Vu08 and five genes were found on Vu11 within the regions of interest. Among these were a gene encoding X8 protein domain (*Vigun08g193600*), an acyltransferases gene (*Vigun08g193700*), a gene encoding Ethylene-responsive protein kinase *Le-CTR1* (*Vigun11g088200*)*,* and an F-box family protein gene (*Vigun11g088300*). The X8 protein domain contains signal sequences that can bridge to the extracellular face of the plasma membrane, and has been reported in carbohydrate binding process in *Arabidopsis* (Simpson et al., 2009).

Vegetable cowpea is known for its long pod length, which is a measure of increased organ size, a fundamental target during domestication that involved many biological processes including cellular metabolism (Lo et al., 2018). This trait is therefore likely to be under quantitative control. Using the current cowpea genome for alignment of SNPs discovered during GBS, and any genomic regions listed from similar genotyping, we found likely domestication QTLs. Identification of flanking QTLs within regions under selection may be biased by the ability to integrate previous mapping based on shared SNP markers between GBS studies, versus others using iSelect arrays.

Meanwhile, floral scent QTL were found to be distinguishing genes in the comparison of vegetable and grain cowpeas. This may be relevant to the movement of germplasm from Africa, where it was co-adapted with pollinators, to Asia, where perhaps pollinators used by *Vigna unguiculata *spp. *sesquipedelis* might not have been abundant. In plants, acyltransferases are members of the large BAHD family, which are involved in floral volatile ester biosynthesis (D’Auria, 2006).

Floral scent facilitates plant-pollinator interaction, which is a domestication syndrome trait in cowpea (Andargie et al., 2014). The F-box genes play an important role in a variety of biological process in plant growth and development (Yang et al., 2008), and have been found to be associated with flower scent in cowpea (Lo et al., 2020). In *Arabidopsis*, the ortholog genes of *Vigun11g088200* and *Vigun08g215300* are *CTR1* and *SNEEZY* respectively, both are important hormone regulators involved in plant growth and development (Li et al., 2001; Leclercq et al., 2002). Therefore, the alleles in the vegetable types for these genes should be analyzed for pollinator attraction.

Seed coat pattern QTLs overlap with a total of seven outlier divergent regions on Vu09 and Vu10 (Herniter et al., 2019). Two genes involved in flavonoid biosynthesis pathway, *Vigun09g139900* and *Vigun10g163900*, have been identified as candidate genes for the development of Holstein seed coat pattern, but none of them located within the overlapped region, likely due to the stringent threshold used.

Due to the mosaic of intermingled divergence signatures, we recommend that future studies use various summary statistics to explore relative differentiation (*F*_*ST*_) as we have done. Looking for spurious concurrent signals through LD analysis, is a straightforward option. Alternatively, comparing the *F*_*ST*_ score with a measure of absolute divergence (*D*_*XY*_) can also inform population differentiation in the presence of gene flow (co-occurrence of peaks in both profiles), recurrent selection across subpopulations (co-occurrence of *F*_*ST*_ peaks with shallow *D*_*XY*_ valleys), or selective sweeps predating the subpopulations’ split (co-occurrence of *F*_*ST*_ peaks with deep *D*_*XY*_ valleys) (Irwin et al., 2016).

Interpreting divergence within a genomic context also helps to overcome a second major bottleneck, which is that the landscape of divergence may miss some well know QTLs of traits putatively under divergent selection. This paradox is partly because QTL/GWAS mapping and GWSS usually look at different underlying processes. The former focuses on statistical associations as a function of recombination and LD (Tam et al., 2019), while the latter aims capturing signatures of divergent (at the meta-population) or directional (at a single population) selection (Cortés et al., 2020). Additionally, identification of flanking QTLs within regions under selection may be biased to the availability of previous reports on QTL mapping for putative target traits.

Researchers are advised to always consider that domestication and crop improvement differ in the traits, strength and timing of selection. Our work here focuses on deep signatures of selection (i.e., signatures of domestication) observable in landraces and older varieties of cowpeas and yardlong beans with differentiation across *FST* profiles enriched after many generations under selection (Cortés et al., 2018). Therefore, it is not uncommon that associations are detected in the absence of notorious selection signatures, depending on the biological nature of the footprint. This was notable in the case of pod type / yardlong bean comparisons with grain and fodder cowpeas, the former of which seem to be derived from. Enough germplasm of spp. *unguiculata* and spp. *sesquipedalis* can be found for continued study. However, lower germplasm availability of spp. *cylindrica* may hamper comparisons with it.

Finally, if more recent selection events (*e.g.*, as part of modern plant improvement initiatives) are of interest, breeding programs in yardlong beans and grain cowpea can be tapped for similar investigation; however with the caveat that breeding lines may have not reached *F*_*ST*_ fixation yet, especially if associated with soft selection sweeps. Therefore, future studies must envision bringing more germplasm concretely into consideration to identify recent selection signatures from breeding work. Existing variation of modern cowpeas has been bred across many national breeding stations so is publicly accessible, but assembly will sometimes be an issue. Meanwhile, horticultural yardlong beans have been bred across fewer programs but are of lower accessibility because they are of private sector interest.

## Conclusion

This study generated a large SNP dataset which we used to reveal the genetic distinction among three cultivated cowpea subspecies through the use of GBS analysis, the newly released cowpea genomic sequence, and collection of diverse pod vegetable, grain and fodder genotypes. The highly informative SNP loci distinguished population structure and relationships among the three subspecies groups, as well as their relationship to a wild species outgroup. A close relationship was revealed between ssp. *unguiculata* and ssp. *cylindrica*, while ssp. *sesquipedalis* shows very distinctive genetic makeup. Discrete signatures of selection and divergence, especially between the pod vegetable type and the other two grain and fodder types were found on chromosome Vu03 and Vu08. These regions of the genome are known to contain candidate genes related to domestication traits, such as pod length, flower scent and seed development. This study has implications on the domestication and selection footprints of cowpea subspecies, and their relationships to each other, especially with the pod types compared to the grain types. The results of this study provide a basis for further fine mapping of genes involved in cowpea domestication, and a genetic foundation for the utilization and exploitation of cowpea germplasms for breeding programs. While we found evidence for ancient selection events leading to the development of yardlong beans as a vegetable derivative from the more agronomic grain cowpeas, with perhaps some influence of fodder types; we did not uncover differentiation between fodder and grain subspecies. More recent selection events (*e.g.*, as part of modern plant improvement initiatives) may have not reached *F*_*ST*_ fixation yet, especially if associated with soft selections sweeps. Therefore, future studies must bring into consideration recent selection signatures on standing variation in all three types of cowpeas if separate germplasm collections are surveyed, especially within the spectrum from landraces to modern varieties of grain cowpeas and yardlong beans in their corresponding breeding programs.

## Materials and methods

### Plant material

A total of 130 cowpea genotypes from three cultivated subspecies were used in this study (Table S1). Most were landraces with a few breeding lines, with 13 genotypes of a fodder cowpea type from spp. *cylindrica*, 30 vegetable cowpea accessions from spp. *sesquipedalis*, and 85 grain cowpea accessions from spp. *unguiculata*. As an outgroup, we used 2 wild *V. unguiculata* accessions with one each from spp. *dekindtiana* and spp. *pubescens*. A total of 94 cowpeas, including all the vegetable and fodder types were sourced from the United States Department of Agriculture (USDA) germplasm collection in Griffin, GA USA. The remaining 36 grain type and one wild accession were sourced from the University of California, Riverside (UC-R) or the University of California, Davis (UC-D).

### DNA extraction and genotyping by sequencing (GBS)

Six seeds of each cowpea genotypes were disinfected by diluted bleach (1%) for ten minutes and germinated in a petri dish maintained in a dark growth chamber for four days. DNA was extracted from the cotyledonary leaves and the shoot apex of the cultivated or wild cowpea seedlings using DNeasy Plant DNA miniprep kits (Qiagen, Hilden, Germany) according to manufacturer’s instructions. The DNA quality and concentrations were evaluated by 1% agarose gel electrophoresis and by absorbance (A) readings at 260/280 nm wavelengths in a FLUOstar Omega (BMG LABTECH) spectrophotometer. After this evaluation, aliquots of 30 ul of each DNA samples with a concentration of more than 50 ng/μl and a A260/A280 threshold of 1.8 reflecting high quality were lyophilized for genotyping by sequencing (GBS) library preparation.

### GBS library preparation and sequencing

GBS library preparation was carried out with approximately 1.5 μg of DNA at the Institute of Biotechnology, Cornell University. The genome size of *V. unguiculata ssp. unguiculata* is approximately 620 Mb (Lonardi et al., 2019), thus restriction enzyme *Ape*KI was used for genomic DNA digestion as this has worked well for similarly sized small genome species (Elshire et al., 2011) and for cowpea in previous GBS studies (Xiong et al., 2016). Common adaptors and bar code ligation, sample pooling and amplification for sequencing library construction were performed according to protocols described previously (Elshire et al., 2011). Size selection after digestion and ligation was for DNA fragments of approximately 300 bp using magnetic beads. Single-end sequencing of the 95-plex library was performed with Illumina HiSeq 2000 platform (Illumina Inc. San Diego, CA, United States) at the Institute of Biotechnology of Cornell University.

### Single nucleotide polymorphism (SNP) calling

SNP variants in the resulting sequences were discovered using the GBS v2 pipeline as implemented in TASSEL v5.0 (Glaubitz et al., 2014). Default parameters were used, except minimum tag read counts were adjusted to 3. The recently published genome *Vigna unguiculata* v1.1 from Phytozome was used as reference genome for indexing and aligned with GBS reads. VCFtools (Danecek et al., 2011) was then used to filter SNPs by excluding indels, non-reference alleles, alleles with more than 10% missing data or minor allele frequency (MAF) below 0.05. Filtered VCF file is available upon request to the corresponding author.

### Population structure and phylogeny analyses

Two steps were conducted to perform a structure analysis. First, genetic principal component analysis (PCA) was performed by TASSEL and visualized using R *ggplot2* package. Subsequently, the number of sub-populations was estimated based on an unsupervised Bayesian clustering algorithm named STRUCTURE (v2.3.4) (Hubisz et al., 2009), with *K* values defined between 2 and 9 by repeated analyses using 10 repetitions without *a* *priori* group assignments. This allowed us to definitively determine the optimal sub-population number and to group each genotype into the most appropriate sub-population using an admixture model with correlated allele frequencies, 20,000 iterations for burn-in, and 20,000 MCMC repetitions for parameter estimation. Analysis output was summarized using the Cluster Markov Packager Across *K* algorithm (CLUMPAK) (Kopelman et al., 2015), and was permuted using a Large *K* Greedy algorithm with random input order and 2,000 repeats. CLUMPP was used to align the multiple runs of genotype clustering to confirm the best K value using the *ΔK* method (Evanno et al., 2005). Membership coefficients for individuals and population structure were visualized with DISTRUCT (Rosenberg, 2004).

A phylogeny was generated with MEGA v6 (Tamura et al., 2013) using a maximum likelihood (ML) reconstruction approach with 500 bootstrap replications. All SNP sites were considered in the model and algorithm parameters included nucleotide substitution based on the general time reversible (GTR) model, gamma distributed mutation rates among sites, and four discrete gamma categories for invariant sites. The maximum likelihood heuristic used nearest-neighbor interchange with a moderate branch swap filter. The phylogeny was anchored by an outgroup clade consisting of two wild cowpea relatives, PI 449218 (*V. u.* ssp. *pubescens*) and UCR 5301 (*V. u.* ssp. *dekindtiana*). They are considered wild subspecies controls, as they are non-cultivated and not directly related to cultivated subspecies: spp. *cylindrica*, *sesquipedalis* and *unguiculata*, and are therefore useful for measuring phylogenetic relationships among the cowpea subspecies.

### Genomic diversity and landscape of divergence among three cowpea subspecies

As a proxy for relative differentiation, we computed the fixation index (*F*_*ST*_) from (Weir and Cockerham 1984) between three groups of cowpea accessions based on their subspecies assignment (spp. *unguiculata*, ssp. *sesquipedalis* and spp. *cylindrica*) with VCFtools, setting a sliding window of 100 kb with a step size of 10 kb, following previous linkage disequilibrium estimates and genomic windowed scans in other legumes. Genomic windows with the top of *F*_*ST*_ values were selected as candidate regions for further analysis. Then, we performed whole-genome permutation tests to ascertain the α = 0.001 thresholds for identifying genomic windows highly differentiated between each of the subgroups. This was done to verify the empirical cutoff for grouping while maintaining a low false discovery rate. Subsequently, nucleotide diversity and linkage disequilibrium (LD), as respectively measured by π (Nei, 1987) and *R*^*2*^ (Slatkin, 2008), were estimated along the whole genome in 100 kb windows with a step size of 10 kb using VCFtools, to better interpret the results of the *F*_*ST*_ analysis and clarify how dry grain, fodder and vegetable genotypes differentiated. Specifically, comparing relative differentiation with estimates of LD allow for concrete inferences on the processes giving rise to the divergence patterns, such as genuine divergent selection (i.e. when divergence opposes LD (Cortés et al., 2018)), or spurious concurrent signals due to suppressed recombination, eroded effective population size (*Ne*), genetic drift (Ellegren and Wolf, 2017) and genomic constrains (Wolf and Ellegren, 2017).

The cowpea genome, *Vigna unguiculata* v1.1, and its list of annotated genes, as found in the Phytozome V12.1 database hosted at https://phytozome.jgi.doe.gov/pz/portal.html, was used to investigate the putative gene functions of the genomic regions with the top 0.1% of *F*_*ST*_ values. In addition, the genes in the regions of putative divergence were categorized based on their different biological processes using R package *topGO32*.

### Supplementary Information

**Additional file 1: Table S1.** Passport data of cowpea accessions used in this study. **Table S2.** Genes discovered within the selective sweep regions between three cowpea subspecies. **Figure S1.** Distribution of genotyping by sequencing (GBS) identified single nucleotide polymorphism (SNP). Marks represent loci found on eleven chromosomes of the *Vigna unguiculata* (IT97K-499-35) cowpea reference genome. Scales at the margins of this figure represent sequence length in mega base-pairs. **Figure S2**. Frequency distributions of relative differentiation (*F*_*ST*_) profiles among three cowpea subspecies. Average pairwise *F*_*ST*_ values per sliding window (window size = 100 kb, step size = 10 kb) are shown between: a) grain and fodder cowpea, b) grain and yardlong bean, and c) yardlong and grain cowpea. Dashed vertical lines mark the α = 0.001 threshold for detection of outliers based on whole-genome permutation tests. **Figure S3.** Genomic landscape of divergence among three cowpea subspecies. Sliding window analyses (window size = 100 kb, step size = 10 kb) are depicted for average relative differentiation (*F*_*ST*_) between: a) grain and fodder cowpea, b) grain and yardlong bean, and c) yardlong and grain cowpea. D, average windowed LD, as measured by *R*^*2*^, for all accessions. Dashed horizontal lines indicate overall mean and the α = 0.001 threshold (upper line, when applicable) in b) and c) for detection of outliers based on whole-genome permutation tests. Green and blue blocks indicate the outlier regions identified between each comparison. Colored dots above the outlier regions in b) and c) mark known QTLs (Table 2) for pod length (in green), flower scent (in red), and seed coat pattern (in blue). Results of all windowed analyses are plotted against window midpoints in millions of base pairs (Mb). *X*-axis always shows physical distance in Mb across the 11 chromosomes of cowpea while the *Y*-axis comparisons always indicate *F*_*st*_ value between C (fodder types), G (grain types), and Y (yardlong vegetable types). **Figure S4.** Nucleotide diversity (π) of three cowpea types. These divided are by a) overall nucleotide diversity, b) fodder type diversity, c) grain type diversity, yardlong vegetable type diversity, and d) average LD across windows, as measured by *R*^*2*^ for all accessions.

## Data Availability

Filtered VCF file is available upon request to the corresponding author.

## Abbreviations

GBS: Genotyping-by-Sequencing
GP: Genomic Prediction
GWAS: Genome Wide Association Studie
GWSS: Genome Wide Selection Scans
IITA: International Institute of Tropical Agriculture
SNP: Single Nucleotide Polymorphism
