# Supplementary material for: Genetic differentiation of grain, fodder and pod vegetable type cowpeas (Vigna unguiculata L.) identified through single nucleotide polymorphisms from genotyping-by-sequencing
Source: Mol Hortic. 2022 Mar 28;2:8. doi: 10.1186/s43897-022-00028-x (PMC10514946; doi:10.1186/s43897-022-00028-x)
Supplement: Supplementary file 1 — Additional file 1: Table S1. Passport data of cowpea accessions used in this study. Table S2. Genes discovered within the selective sweep regions between three cowpea subspecies. Figure S1. Distribution of genotyping by sequencing (GBS) identified single nucleotide polymorphism (SNP). Marks represent loci found on eleven chromosomes of the Vigna unguiculata (IT97K-499-35) cowpea reference genome. Scales at the margins of this figure represent sequence length in mega base-pairs. Figure S2. Frequency distributions of relative differentiation (FST) profiles among three cowpea subspecies. Average pairwise FST values per sliding window (window size = 100 kb, step size = 10 kb) are shown between: a) grain and fodder cowpea, b) grain and yardlong bean, and c) yardlong and grain cowpea. Dashed vertical lines mark the α = 0.001 threshold for detection of outliers based on whole-genome permutation tests. Figure S3. Genomic landscape of divergence among three cowpea subspecies. Sliding window analyses (window size = 100 kb, step size = 10 kb) are depicted for average relative differentiation (FST) between: a) grain and fodder cowpea, b) grain and yardlong bean, and c) yardlong and grain cowpea. D, average windowed LD, as measured by R2, for all accessions. Dashed horizontal lines indicate overall mean and the α = 0.001 threshold (upper line, when applicable) in b) and c) for detection of outliers based on whole-genome permutation tests. Green and blue blocks indicate the outlier regions identified between each comparison. Colored dots above the outlier regions in b) and c) mark known QTLs (Table 2) for pod length (in green), flower scent (in red), and seed coat pattern (in blue). Results of all windowed analyses are plotted against window midpoints in millions of base pairs (Mb). X-axis always shows physical distance in Mb across the 11 chromosomes of cowpea while the Y-axis comparisons always indicate Fst value between C (fodder types), G (grain types), and Y (yardlong vegeta [file 43897_2022_28_MOESM1_ESM.pdf]

Table S1. Passport data of cowpea accessions used in this study.

Code	Accession	Name	Taxa	Origin	Source	Cluster membership	
						K=2	K=4
11	PI 184952	TVu 1547	*V. unguiculata* ssp. *unguiculata*	Ghana	USDA-Grin	1	2
14	PI 186460	TVu 1551	*V. unguiculata* ssp. *unguiculata*	Nigeria	USDA-Grin	1	2
15	PI 186465	TVu 1950	*V. unguiculata* ssp. *unguiculata*	Nigeria	USDA-Grin	1	2
18	PI 189375	TVu 1738	*V. unguiculata* ssp. *unguiculata*	Nigeria	USDA-Grin	1	2
19	PI 189378	TVu 1692	*V. unguiculata* ssp. *unguiculata*	Nigeria	USDA-Grin	1	2
48	PI 255774	TVu 2428	*V. unguiculata* ssp. *unguiculata*	Nigeria	USDA-Grin	1	2
49	PI 255782	TVu 1956	*V. unguiculata* ssp. *unguiculata*	Nigeria	USDA-Grin	admix	admix
50	PI 255811	TVu 1958	*V. unguiculata* ssp. *unguiculata*	Nigeria	USDA-Grin	1	2
51	PI 255815	TVu 1459	*V. unguiculata* ssp. *unguiculata*	Nigeria	USDA-Grin	1	2
145	PI 447582	TVu 3834	*V. unguiculata* ssp. *unguiculata*	Nigeria	USDA-Grin	1	1
146	PI 448112	TVu 4776	*V. unguiculata* ssp. *unguiculata*	Niger	USDA-Grin	1	1
147	PI 448144	TVu 4811	*V. unguiculata* ssp. *unguiculata*	Niger	USDA-Grin	1	1
148	PI 448192	TVu 4862	*V. unguiculata* ssp. *unguiculata*	Niger	USDA-Grin	1	1
161	PI 580623	TVu 7907	*V. unguiculata* ssp. *unguiculata*	Nigeria	USDA-Grin	1	2
162	PI 580867	TVu 10660	*V. unguiculata* ssp. *unguiculata*	Nigeria	USDA-Grin	1	1
171	PI 582419	KVu 69	*V. unguiculata* ssp. *unguiculata*	Burkina Faso	USDA-Grin	1	admix
174	PI 582512	UCR 430	*V. unguiculata* ssp. *unguiculata*	Nigeria	USDA-Grin	admix	admix
175	PI 582530	SAMBRIZIE	*V. unguiculata* ssp. *unguiculata*	Ghana	USDA-Grin	1	2
207	PI 582922	UCR 53	*V. unguiculata* ssp. *unguiculata*	Burkina Faso	USDA-Grin	1	2
214	PI 582951	CAMEROON-2	*V. unguiculata* ssp. *unguiculata*	Cameroon	USDA-Grin	1	admix
215	PI 582952	UCR 452	*V. unguiculata* ssp. *unguiculata*	Nigeria	USDA-Grin	1	1
222	PI 583096	TOULOUM 01	*V. unguiculata* ssp. *unguiculata*	Cameroon	USDA-Grin	1	1
223	PI 583100	UCR 2620	*V. unguiculata* ssp. *unguiculata*	Cameroon	USDA-Grin	1	admix
224	PI 583102	UCR 2623	*V. unguiculata* ssp. *unguiculata*	Cameroon	USDA-Grin	1	admix
225	PI 583104	UCR 2625	*V. unguiculata* ssp. *unguiculata*	Cameroon	USDA-Grin	1	admix
226	PI 583106	UCR 2627	*V. unguiculata* ssp. *unguiculata*	Cameroon	USDA-Grin	admix	admix
227	PI 583108	UCR 2631	*V. unguiculata* ssp. *unguiculata*	Cameroon	USDA-Grin	1	admix
229	PI 583112	UCR 2637	*V. unguiculata* ssp. *unguiculata*	Cameroon	USDA-Grin	1	1
230	PI 583154	UCR 2695	*V. unguiculata* ssp. *unguiculata*	Cameroon	USDA-Grin	1	1
231	PI 583160	UCR 2702	*V. unguiculata* ssp. *unguiculata*	Cameroon	USDA-Grin	1	admix
232	PI 583164	UCR 2708	*V. unguiculata* ssp. *unguiculata*	Cameroon	USDA-Grin	1	admix
233	PI 583167	UCR 2713	*V. unguiculata* ssp. *unguiculata*	Cameroon	USDA-Grin	admix	admix
234	PI 583170	UCR 2716	*V. unguiculata* ssp. *unguiculata*	Cameroon	USDA-Grin	1	admix
238	PI 583195	UCR 3338	*V. unguiculata* ssp. *unguiculata*	Senegal	USDA-Grin	admix	admix
243	PI 583219	UCR 3285	*V. unguiculata* ssp. *unguiculata*	Senegal	USDA-Grin	1	admix
251	PI 583232	UCR 3317	*V. unguiculata* ssp. *unguiculata*	Senegal	USDA-Grin	1	admix
254	PI 583236	UCR 3327	*V. unguiculata* ssp. *unguiculata*	Senegal	USDA-Grin	1	1
255	PI 583238	UCR 3339	*V. unguiculata* ssp. *unguiculata*	Senegal	USDA-Grin	1	admix
258	PI 583244	UCR 3360	*V. unguiculata* ssp. *unguiculata*	Senegal	USDA-Grin	1	admix
260	PI 583246	UCR 3365	*V. unguiculata* ssp. *unguiculata*	Senegal	USDA-Grin	1	1
265	PI 583258	UCR 178	*V. unguiculata* ssp. *unguiculata*	Senegal	USDA-Grin	1	admix

284	PI 448427	TVu 5115	*V. unguiculata* ssp. *unguiculata*	Niger	USDA-Grin	1	1
285	PI 448499	TVu 5247	*V. unguiculata* ssp. *unguiculata*	Niger	USDA-Grin	1	admix
286	PI 448558	TVu 5315	*V. unguiculata* ssp. *unguiculata*	Niger	USDA-Grin	1	1
288	PI 448702	TVu 5493	*V. unguiculata* ssp. *unguiculata*	Niger	USDA-Grin	1	1
290	PI 448806	TVu 5616	*V. unguiculata* ssp. *unguiculata*	Niger	USDA-Grin	1	1
291	PI 449161	TVu 6949	*V. unguiculata* ssp. *unguiculata*	Niger	USDA-Grin	1	1
292	PI 449218	TVu 7006	*V. unguiculata* ssp. *unguiculata*	Niger	USDA-Grin	1	1
294	PI 582531	SUMBRISOGLA	*V. unguiculata* ssp. *unguiculata*	Ghana	USDA-Grin	1	2
297	PI 583098	UCR 2617	*V. unguiculata* ssp. *unguiculata*	Cameroon	USDA-Grin	1	admix
298	PI 583156	UCR 2698	*V. unguiculata* ssp. *unguiculata*	Cameroon	USDA-Grin	1	admix
300	PI 406365	TVnu 112	*V. unguiculata* ssp. *pubescens*	Tanzania	USDA-Grin	admix	admix
C01	PI 291384	No.276	*V. unguiculata* ssp. *cylindrica*	China	USDA-Grin	2	admix
C02	PI 189416	51-50	*V. unguiculata* ssp. *cylindrica*	Guatemala	USDA-Grin	admix	admix
C03	PI 304164	A-269	*V. unguiculata* ssp. *cylindrica*	Honduras	USDA-Grin	2	3
C04	PI 205140		*V. unguiculata* ssp. *cylindrica*	India	USDA-Grin	admix	admix
C05	PI 205139		*V. unguiculata* ssp. *cylindrica*	India	USDA-Grin	admix	admix
C06	PI 255755		*V. unguiculata* ssp. *cylindrica*	Nigeria	USDA-Grin	1	2
C07	PI 250238	K522	*V. unguiculata* ssp. *cylindrica*	Pakistan	USDA-Grin	admix	admix
C08	PI 270065	RAMBO	*V. unguiculata* ssp. *cylindrica*	Pakistan	USDA-Grin	admix	admix
C09	PI 180494	10841	*V. unguiculata* ssp. *cylindrica*	India	USDA-Grin	admix	admix
C10	PI 180355	CHAWLI	*V. unguiculata* ssp. *cylindrica*	India	USDA-Grin	admix	admix
C11	PI 180014	CHOLAN	*V. unguiculata* ssp. *cylindrica*	India	USDA-Grin	admix	admix
C12	PI 201498	7124	*V. unguiculata* ssp. *cylindrica*	Mexico	USDA-Grin	admix	admix
C13	PI 167284	BRABHAM	*V. unguiculata* ssp. *cylindrica*	Turkey	USDA-Grin	2	3
Y01	PI 146618	TVu 1411	*V. unguiculata* ssp. *sesquipedalis*	Brazil	USDA-Grin	2	4
Y02	PI 147071	No.1	*V. unguiculata* ssp. *sesquipedalis*	Brazil	USDA-Grin	2	4
Y03	PI 196301	2925	*V. unguiculata* ssp. *sesquipedalis*	Nicaragua	USDA-Grin	2	4
Y04	PI 215659	PHILIPPINE EARLY	*V. unguiculata* ssp. *sesquipedalis*	India	USDA-Grin	2	4
Y05	PI 271258	TVu 2449	*V. unguiculata* ssp. *sesquipedalis*	India	USDA-Grin	2	4
Y06	PI 353045	TVu 2891	*V. unguiculata* ssp. *sesquipedalis*	India	USDA-Grin	2	4
Y07	PI 353074	UCR 2576	*V. unguiculata* ssp. *sesquipedalis*	India	USDA-Grin	2	4
Y08	PI 406985	W-C 1559	*V. unguiculata* ssp. *sesquipedalis*	Nicaragua	USDA-Grin	2	4
Y10	PI 419102		*V. unguiculata* ssp. *sesquipedalis*	China	USDA-Grin	2	4
Y11	PI 419165	YI TIAN HONG 752	*V. unguiculata* ssp. *sesquipedalis*	China	USDA-Grin	2	4
Y12	PI 430687	HUA PI CHIA CHIANG TOU	*V. unguiculata* ssp. *sesquipedalis*	China	USDA-Grin	2	4
Y13	PI 478396	O 112	*V. unguiculata* ssp. *sesquipedalis*	China	USDA-Grin	2	4
Y14	PI 487486	Dagupan Pangasinan Collection No. 1.3	*V. unguiculata* ssp. *sesquipedalis*	Philippines	USDA-Grin	2	4
Y15	PI 487502	ECO CAR POLE Sitao #2	*V. unguiculata* ssp. *sesquipedalis*	Philippines	USDA-Grin	2	4
Y16	PI 487507	Dagupan Pangasinan Collection No. 3.1	*V. unguiculata* ssp. *sesquipedalis*	Philippines	USDA-Grin	2	4
Y17	PI 487516	Bush Sitao	*V. unguiculata* ssp. *sesquipedalis*	Philippines	USDA-Grin	2	4
Y18	PI 487519	Tainong's 1	*V. unguiculata* ssp. *sesquipedalis*	Taiwan	USDA-Grin	2	4
Y19	PI 487527	K-30	*V. unguiculata* ssp. *sesquipedalis*	United States	USDA-Grin	2	4
Y20	PI 487532	CP 55	*V. unguiculata* ssp. *sesquipedalis*	Indonesia	USDA-Grin	2	4
Y21	PI 487534	CP 57	*V. unguiculata* ssp. *sesquipedalis*	Taiwan	USDA-Grin	2	4
Y22	PI 487539	CP 63	*V. unguiculata* ssp. *sesquipedalis*	Philippines	USDA-Grin	2	4

Y23	PI 487549	CP 73	*V. unguiculata* ssp. *sesquipedalis*	Indonesia	USDA-Grin	2	4
Y24	PI 487550	CP 74	*V. unguiculata* ssp. *sesquipedalis*	Taiwan	USDA-Grin	2	4
Y25	PI 490770	Huang Hua Qing Ai Jiang Dou	*V. unguiculata* ssp. *sesquipedalis*	China	USDA-Grin	2	4
Y26	PI 578902	CHANG XIAN JIANG DOU	*V. unguiculata* ssp. *sesquipedalis*	China	USDA-Grin	2	4
Y27	PI 419005		*V. unguiculata* ssp. *sesquipedalis*	China	USDA-Grin	2	4
Y28	PI 487523	Tianong's 5	*V. unguiculata* ssp. *sesquipedalis*	China	USDA-Grin	2	4
Y29	PI 468104	PRT 4	*V. unguiculata* ssp. *sesquipedalis*	Suriname	USDA-Grin	2	4
R01	PI 148674	TVu 1522	*V. unguiculata* ssp. *unguiculata*	Iran	UC-Riverside	2	3
R02	DAN Iia	DAN Ila	*V. unguiculata* ssp. *unguiculata*	IITA Breeding line	UC-Riverside	1	1
R04	PI 582879	UCR 1115	*V. unguiculata* ssp. *unguiculata*	Botswana	UC-Riverside	admix	2
R05	TVu 7778	TVu 7778	*V. unguiculata* ssp. *unguiculata*	Côte d'Ivoire	UC-Riverside	1	2
R06	PI 418979	HAN CHUI YEN	*V. unguiculata* ssp. *sesquipedalis*	China	UC-Riverside	2	4
R07	PI 582557	UCR 372	*V. unguiculata* ssp. *unguiculata*	Kenya	UC-Riverside	admix	admix
R08	524B	524B	*V. unguiculata* ssp. *unguiculata*	UCR Breeding line MP	UC-Riverside	admix	2
R09	UCR 5301	TVNu-463	*V. unguiculata* ssp. *dekindtiana*	Botswana	UC-Riverside	admix	admix
R11	IT97K-461-4	IT97K-461-4	*V. unguiculata* ssp. *unguiculata*	IITA Breeding line	UC-Riverside	admix	admix
R12	CB46	CB46	*V. unguiculata* ssp. *unguiculata*	UCR Cultivar MP	UC-Riverside	admix	admix
R13	IT982E-18	IT982E-18	*V. unguiculata* ssp. *unguiculata*	IITA Breeding line	UC-Riverside	admix	admix
R15	CB27	CB27	*V. unguiculata* ssp. *unguiculata*	UCR Cultivar MP	UC-Riverside	admix	2
R16	IT84S-2049	IT84S-2049	*V. unguiculata* ssp. *unguiculata*	IITA Breeding line	UC-Riverside	admix	admix
R17	IT93K503-1	IT93K503-1	*V. unguiculata* ssp. *unguiculata*	IITA Breeding line	UC-Riverside	admix	admix
S01	PI 579009	TVu 200/M088	*V. unguiculata* ssp. *unguiculata*	Nigeria	UC-Davis	admix	2
S06	PI 582393	UCR 232/M025	*V. unguiculata* ssp. *unguiculata*	Nigeria	UC-Davis	admix	admix
S07	M101	M101	*V. unguiculata* ssp. *unguiculata*	South Africa	UC-Davis	2	4
S17	PI 579815	TVu 1985/G15	*V. unguiculata* ssp. *unguiculata*	Nigeria	UC-Davis	1	admix
S18	TG296	TVu 14533	*V. unguiculata* ssp. *unguiculata*	Mali	UC-Davis	2	3
S23	CG021	CG021	*V. unguiculata* ssp. *sesquipedalis*	Philippines	UC-Davis	2	4
S24	PI 304150	TVu 1996/M121	*V. unguiculata* ssp. *unguiculata*	Nicaragua	UC-Davis	2	3
S25	TG198	TVu 9234	*V. unguiculata* ssp. *unguiculata*	Tanzania	UC-Davis	2	3
S26	PI 583550	UCR 4660/CG033	*V. unguiculata* ssp. *unguiculata*	Mali	UC-Davis	admix	admix
S27	TG209	TVu 9474	*V. unguiculata* ssp. *unguiculata*	Egypt	UC-Davis	admix	admix
S28	PI 583494	UCR 4547/CG031	*V. unguiculata* ssp. *unguiculata*	Nigeria	UC-Davis	1	admix
S29	TG208	TVu 9469	*V. unguiculata* ssp. *unguiculata*	Egypt	UC-Davis	2	3
S30	TG167	TVu 7991	*V. unguiculata* ssp. *unguiculata*	Nigeria	UC-Davis	admix	admix
S31	TG212	TVu 9506	*V. unguiculata* ssp. *unguiculata*	Egypt	UC-Davis	admix	admix
S32	PI 447579	TVu 3830/TG084	*V. unguiculata* ssp. *unguiculata*	Nigeria	UC-Davis	admix	admix
S33	PI 580290	TG150/TVU-7127	*V. unguiculata* ssp. *unguiculata*	Nigeria	UC-Davis	2	admix
S05	PI 583259	SUVITA 2/M015	*V. unguiculata* ssp. *unguiculata*	Burkina Faso	UC-Davis	1	1
S08	TG229	TVu 10179	*V. unguiculata* ssp. *unguiculata*	India	UC-Davis	admix	admix
S10	TG102	TVu 4760	*V. unguiculata* ssp. *unguiculata*	IITA	UC-Davis	1	2
S11	TG184	TVu 8713	*V. unguiculata* ssp. *unguiculata*	Benin	UC-Davis	1	2
S13	TG137	TVu 6644	*V. unguiculata* ssp. *unguiculata*	Liberia	UC-Davis	2	4
S16	TG274	TVu 13939	*V. unguiculata* ssp. *unguiculata*	Botswana	UC-Davis	admix	admix
S19	PI 447634	TVu 3890/CG065	*V. unguiculata* ssp. *unguiculata*	Nigeria	UC-Davis	1	admix

Table S2. Genes discovered within the selective sweep regions between three cowpea subspecies.

Region*	Flanking Search	Gene Name	Chromosome	Start (bp)	End (bp)	PFAM	Description (top hit)
1	Vu02:24552281..24652281	*Vigun02g090700*	Vu02	24571133	24573755	PFNANANA1NA	Helix-loop-helix DNA-binding domain
1	Vu02:24552281..24652281	*Vigun02g090800*	Vu02	24587248	24590667	PFNA1412	Putative GTPase activating protein for Arf
1	Vu02:24552281..24652281	*Vigun02g090900*	Vu02	24598079	24602788	PF13423	Ubiquitin carboxyl-terminal hydrolase
1	Vu02:24552281..24652281	*Vigun02g091000*	Vu02	24609039	24611251	NA	NA
1	Vu02:24552281..24652281	*Vigun02g091100*	Vu02	24612303	24613111	NA	NA
1	Vu02:24552281..24652281	*Vigun02g091200*	Vu02	24620634	24621651	NA	NA
1	Vu02:24552281..24652281	*Vigun02g091300*	Vu02	24621765	24622283	PFNA6NA94	AIG2-like family
1	Vu02:24552281..24652281	*Vigun02g091400*	Vu02	24636639	24638013	PFNA534NA	Protein of unknown function (DUF74NA)
1	Vu02:24552281..24652281	*Vigun02g091500*	Vu02	24640249	24641209	NA	NA
2	Vu03:7383261..7483261	*Vigun03g088200*	Vu03	7405865	7409219	PF14365	Domain of unknown function (DUF44NA9)
2	Vu03:7383261..7483261	*Vigun03g088300*	Vu03	7428684	7431589	NA	NA
2	Vu03:7383261..7483261	*Vigun03g088400*	Vu03	7433202	7433769	NA	NA
2	Vu03:7383261..7483261	*Vigun03g088500*	Vu03	7436167	7440132	PFNANANA67	Cytochrome P45NA
2	Vu03:7383261..7483261	*Vigun03g088600*	Vu03	7440574	7443421	NA	NA
2	Vu03:7383261..7483261	*Vigun03g088700*	Vu03	7444129	7450371	PFNA7714	Protein tyrosine kinase
2	Vu03:7383261..7483261	*Vigun03g088800*	Vu03	7456096	7460356	PF14432	DYW family of nucleic acid deaminases
2	Vu03:7383261..7483261	*Vigun03g088900*	Vu03	7461470	7462549	PFNA4641	Rtf2 RING-finger
2	Vu03:7383261..7483261	*Vigun03g089000*	Vu03	7470534	7474654	PFNA4146	YT521-B-like domain
2	Vu03:7383261..7483261	*Vigun03g089100*	Vu03	7474967	7480864	PF1NA241	Uncharacterized conserved protein
2	Vu03:7383261..7483261	*Vigun03g089200*	Vu03	7481704	7485869	PF14531	Kinase-like
3	Vu03:13496721..13596721	*Vigun03g137600*	Vu03	13492033	13507367	PF13NA87	AAA domain
3	Vu03:13496721..13596721	*Vigun03g137700*	Vu03	13523803	13525316	NA	NA
3	Vu03:13496721..13596721	*Vigun03g137800*	Vu03	13539068	13542409	PF12854	PPR repeat
3	Vu03:13496721..13596721	*Vigun03g137900*	Vu03	13543244	13545273	NA	NA
3	Vu03:13496721..13596721	*Vigun03g138000*	Vu03	13545883	13548591	PFNANA995	Sec1 family
3	Vu03:13496721..13596721	*Vigun03g138100*	Vu03	13549562	13549747	NA	NA
3	Vu03:13496721..13596721	*Vigun03g138200*	Vu03	13567448	13571654	NA	NA
3	Vu03:13496721..13596721	*Vigun03g138300*	Vu03	13585398	13588087	PFNA31NA6	WRKY DNA -binding domain
3	Vu03:13496721..13596721	*Vigun03g138400*	Vu03	13595882	13601098	PFNANANA69	Protein kinase domain
4	Vu03:13569671..13669671	*Vigun03g138200*	Vu03	13567448	13571654	NA	NA
4	Vu03:13569671..13669671	*Vigun03g138300*	Vu03	13585398	13588087	PFNA31NA6	WRKY DNA -binding domain
4	Vu03:13569671..13669671	*Vigun03g138400*	Vu03	13595882	13601098	PFNANANA69	Protein kinase domain
4	Vu03:13569671..13669671	*Vigun03g138500*	Vu03	13603169	13608272	PFNA78NANA	Protein of unknown function (DUF1644)
4	Vu03:13569671..13669671	*Vigun03g138600*	Vu03	13618833	13620768	PFNA4852	Protein of unknown function (DUF64NA)
4	Vu03:13569671..13669671	*Vigun03g138700*	Vu03	13639314	13646906	PFNANANA69	Protein kinase domain

4	Vu03:13569671..13669671	*Vigun03g138800*	Vu03	13669521	13673653	PFNA31NA6	WRKY DNA -binding domain
5	Vu03:13735361..13835361	*Vigun03g139400*	Vu03	13734764	13736868	PFNA3145	Seven in absentia protein family
5	Vu03:13735361..13835361	*Vigun03g139500*	Vu03	13737463	13738512	NA	NA
5	Vu03:13735361..13835361	*Vigun03g139600*	Vu03	13785300	13793564	PF12796	Ankyrin repeats (3 copies)
5	Vu03:13735361..13835361	*Vigun03g139700*	Vu03	13803938	13806823	PF13962	Domain of unknown function
5	Vu03:13735361..13835361	*Vigun03g139800*	Vu03	13818502	13820778	PFNA8NA31	Berberine and berberine like
5	Vu03:13735361..13835361	*Vigun03g139900*	Vu03	13822684	13825459	NA	NA
6	Vu03:13743301..13843301	*Vigun03g139600*	Vu03	13785300	13793564	PF12796	Ankyrin repeats (3 copies)
6	Vu03:13743301..13843301	*Vigun03g139700*	Vu03	13803938	13806823	PF13962	Domain of unknown function
6	Vu03:13743301..13843301	*Vigun03g139800*	Vu03	13818502	13820778	PFNA8NA31	Berberine and berberine like
6	Vu03:13743301..13843301	*Vigun03g139900*	Vu03	13822684	13825459	NA	NA
7	Vu03:13745501..13845501	*Vigun03g139600*	Vu03	13785300	13793564	PF12796	Ankyrin repeats (3 copies)
7	Vu03:13745501..13845501	*Vigun03g139700*	Vu03	13803938	13806823	PF13962	Domain of unknown function
7	Vu03:13745501..13845501	*Vigun03g139800*	Vu03	13818502	13820778	PFNA8NA31	Berberine and berberine like
7	Vu03:13745501..13845501	*Vigun03g139900*	Vu03	13822684	13825459	NA	NA
8	Vu03:14339911..14439911	*Vigun03g142200*	Vu03	14342625	14344235	PFNA8NA31	Berberine and berberine like
8	Vu03:14339911..14439911	*Vigun03g142300*	Vu03	14353927	14355558	PFNA8NA31	Berberine and berberine like
8	Vu03:14339911..14439911	*Vigun03g142400*	Vu03	14358927	14360446	PFNA8NA31	Berberine and berberine like
8	Vu03:14339911..14439911	*Vigun03g142500*	Vu03	14385056	14385868	NA	NA
8	Vu03:14339911..14439911	*Vigun03g142600*	Vu03	14389437	14391017	PFNA8NA31	Berberine and berberine like
8	Vu03:14339911..14439911	*Vigun03g142700*	Vu03	14401226	14403170	PFNA8NA31	Berberine and berberine like
8	Vu03:14339911..14439911	*Vigun03g142800*	Vu03	14408879	14411215	NA	NA
8	Vu03:14339911..14439911	*Vigun03g142900*	Vu03	14415393	14417601	PF13812	Pentatricopeptide repeat domain
8	Vu03:14339911..14439911	*Vigun03g143000*	Vu03	14421288	14423936	PFNA8NA31	Berberine and berberine like
8	Vu03:14339911..14439911	*Vigun03g143100*	Vu03	14428040	14429895	PFNA8NA31	Berberine and berberine like
9	Vu03:14521291..14621291	*Vigun03g143600*	Vu03	14520316	14523580	PFNA4116	Fatty acid hydroxylase superfamily
9	Vu03:14521291..14621291	*Vigun03g143700*	Vu03	14539020	14540990	PFNA3953	Tubulin C-terminal domain
9	Vu03:14521291..14621291	*Vigun03g143800*	Vu03	14545834	14553801	PFNA2585	GlcNAc-PI de-N-acetylase
9	Vu03:14521291..14621291	*Vigun03g143900*	Vu03	14560082	14567537	PFNANA481	Protein phosphatase 2C
9	Vu03:14521291..14621291	*Vigun03g144000*	Vu03	14567788	14574941	PFNANA815	Histidinol dehydrogenase
9	Vu03:14521291..14621291	*Vigun03g144100*	Vu03	14590308	14598445	PF13847	Methyltransferase domain
9	Vu03:14521291..14621291	*Vigun03g144200*	Vu03	14602742	14605903	NA	NA
9	Vu03:14521291..14621291	*Vigun03g144300*	Vu03	14620401	14622105	PFNA7712	Stress up-regulated Nod 19
10	Vu04:2848691..2948691	*Vigun04g034500*	Vu04	2855723	2858122	PFNA379NA	KNOX1 domain
10	Vu04:2848691..2948691	*Vigun04g034600*	Vu04	2861323	2864727	PFNA12NA7	Dihydrouridine synthase (Dus)
10	Vu04:2848691..2948691	*Vigun04g034700*	Vu04	2879931	2881227	PF14531	Kinase-like
10	Vu04:2848691..2948691	*Vigun04g034800*	Vu04	2893720	2895713	PF12854	PPR repeat
10	Vu04:2848691..2948691	*Vigun04g034900*	Vu04	2898910	2901500	NA	NA

10	Vu04:2848691..2948691	*Vigun04g035000*	Vu04	2903282	2905638	PFNA3791	KNOX2 domain
10	Vu04:2848691..2948691	*Vigun04g035100*	Vu04	2917051	2921278	NA	NA
10	Vu04:2848691..2948691	*Vigun04g035200*	Vu04	2925977	2927299	PFNA4564	U-box domain
10	Vu04:2848691..2948691	*Vigun04g035300*	Vu04	2935509	2942736	PFNANA5NA3	G-protein alpha subunit
10	Vu04:2848691..2948691	*Vigun04g035400*	Vu04	2945309	2948185	PFNA4722	Ssu72-like protein
11	Vu06:20147641..20247641	*Vigun06g070800*	Vu06	20165315	20165737	NA	NA
11	Vu06:20147641..20247641	*Vigun06g070900*	Vu06	20172264	20173215	NA	NA
11	Vu06:20147641..20247641	*Vigun06g071000*	Vu06	20176074	20176902	NA	NA
11	Vu06:20147641..20247641	*Vigun06g071100*	Vu06	20187978	20189197	NA	NA
11	Vu06:20147641..20247641	*Vigun06g071200*	Vu06	20189426	20190216	NA	NA
11	Vu06:20147641..20247641	*Vigun06g071300*	Vu06	20196730	20198882	PFNA2144	Repair protein Rad1/Rec1/Rad17
11	Vu06:20147641..20247641	*Vigun06g071400*	Vu06	20199501	20209540	PFNANA153	Mitochondrial carrier protein
11	Vu06:20147641..20247641	*Vigun06g071500*	Vu06	20209513	20211790	PFNA4859	Plant protein of unknown function (DUF641)
11	Vu06:20147641..20247641	*Vigun06g071600*	Vu06	20217639	20219929	NA	NA
11	Vu06:20147641..20247641	*Vigun06g071700*	Vu06	20222959	20236575	PFNA8513	LisH
11	Vu06:20147641..20247641	*Vigun06g071800*	Vu06	20239328	20259663	PFNANA271	Helicase conserved C-terminal domain
12	Vu06:22760701..22860701	*Vigun06g096300*	Vu06	22762360	22767015	PF12796	Ankyrin repeats (3 copies)
12	Vu06:22760701..22860701	*Vigun06g096400*	Vu06	22770516	22774078	PFNANA249	Myb-like DNA-binding domain
12	Vu06:22760701..22860701	*Vigun06g096500*	Vu06	22774332	22776139	PFNA333NA	Rare lipoprotein A (RlpA)-like double-psi beta-barrel
12	Vu06:22760701..22860701	*Vigun06g096600*	Vu06	22787075	22796911	PF16457	NA
12	Vu06:22760701..22860701	*Vigun06g096700*	Vu06	22802786	22806626	PFNA2365	No apical meristem (NAM) protein
12	Vu06:22760701..22860701	*Vigun06g096800*	Vu06	22810505	22815288	NA	NA
12	Vu06:22760701..22860701	*Vigun06g096900*	Vu06	22817462	22822116	PFNANA534	Glycosyl transferases group 1
12	Vu06:22760701..22860701	*Vigun06g097000*	Vu06	22826409	22828413	PFNANANA43	Glutathione S-transferase, C-terminal domain
12	Vu06:22760701..22860701	*Vigun06g097100*	Vu06	22838104	22844691	PFNANA282	Pyridoxal-dependent decarboxylase conserved domain
12	Vu06:22760701..22860701	*Vigun06g097200*	Vu06	22845899	22847935	PFNANANA69	Protein kinase domain
12	Vu06:22760701..22860701	*Vigun06g097300*	Vu06	22854568	22856420	PFNA7714	Protein tyrosine kinase
12	Vu06:22760701..22860701	*Vigun06g097400*	Vu06	22859072	22860773	PFNANA646	F-box domain
13	Vu06:23176891..23276891	*Vigun06g101500*	Vu06	23176782	23184245	PFNANA571	CBS domain
13	Vu06:23176891..23276891	*Vigun06g101600*	Vu06	23185102	23190395	PFNANA641	Zn-finger in Ran binding protein and others
13	Vu06:23176891..23276891	*Vigun06g101700*	Vu06	23190983	23196483	PFNA1477	PLAT/LH2 domain
13	Vu06:23176891..23276891	*Vigun06g101800*	Vu06	23203201	23209401	PFNANANA69	Protein kinase domain
13	Vu06:23176891..23276891	*Vigun06g101900*	Vu06	23211477	23212956	NA	NA
13	Vu06:23176891..23276891	*Vigun06g102000*	Vu06	23214086	23221475	PF16188	NA
13	Vu06:23176891..23276891	*Vigun06g102100*	Vu06	23221843	23225004	PF12146	Putative lysophospholipase
13	Vu06:23176891..23276891	*Vigun06g102200*	Vu06	23225718	23228138	PF13459	4Fe-4S single cluster domain
13	Vu06:23176891..23276891	*Vigun06g102300*	Vu06	23228938	23241542	NA	NA
13	Vu06:23176891..23276891	*Vigun06g102400*	Vu06	23247969	23249936	PF14432	DYW family of nucleic acid deaminases

13	Vu06:23176891..23276891	*Vigun06g102500*	Vu06	23250618	23257381	PFNA2836	Glycosyl hydrolases family 2, TIM barrel domain
13	Vu06:23176891..23276891	*Vigun06g102600*	Vu06	23260110	23263204	PFNA15NA1	Glycosyl transferase family 8
13	Vu06:23176891..23276891	*Vigun06g102700*	Vu06	23266218	23267870	PFNANA134	Cyclin, N-terminal domain
13	Vu06:23176891..23276891	*Vigun06g102800*	Vu06	23268669	23271735	PFNA789NA	Rrp15p
13	Vu06:23176891..23276891	*Vigun06g102900*	Vu06	23275424	23279635	PFNA4893	Yip1 domain
14	Vu06:23299871..23399871	*Vigun06g103300*	Vu06	23299530	23303198	PFNA8544	GHMP kinases C terminal
14	Vu06:23299871..23399871	*Vigun06g103400*	Vu06	23304059	23304565	NA	NA
14	Vu06:23299871..23399871	*Vigun06g103500*	Vu06	23305699	23308583	PFNA1535	PPR repeat
14	Vu06:23299871..23399871	*Vigun06g103600*	Vu06	23312826	23315898	PFNANA8NA8	Histone-like transcription factor (CBF/NF-Y) and archaeal histone
14	Vu06:23299871..23399871	*Vigun06g103700*	Vu06	23325775	23328958	PFNA1699	Sodium/calcium exchanger protein
14	Vu06:23299871..23399871	*Vigun06g103800*	Vu06	23329054	23333065	PFNA4573	Signal peptidase subunit
14	Vu06:23299871..23399871	*Vigun06g103900*	Vu06	23335110	23336531	PFNANANA26	Eukaryotic aspartyl protease
14	Vu06:23299871..23399871	*Vigun06g104000*	Vu06	23341624	23343924	PFNANA4NA3	Heavy-metal-associated domain
14	Vu06:23299871..23399871	*Vigun06g104100*	Vu06	23349294	23350755	PF13639	Ring finger domain
14	Vu06:23299871..23399871	*Vigun06g104200*	Vu06	23376676	23378643	PFNANANA1NA	Helix-loop-helix DNA-binding domain
14	Vu06:23299871..23399871	*Vigun06g104300*	Vu06	23388090	23394843	PFNA1NA63	Aminotransferase class IV
14	Vu06:23299871..23399871	*Vigun06g104400*	Vu06	23396310	23399519	PF1NA294	Putative methyltransferase
15	Vu08:32088851..32188851	*Vigun08g148300*	Vu08	32088312	32096129	PFNA1841	Transglutaminase-like superfamily
15	Vu08:32088851..32188851	*Vigun08g148400*	Vu08	32097510	32098988	PFNA6246	Isy1-like splicing family
15	Vu08:32088851..32188851	*Vigun08g148500*	Vu08	32099336	32100835	NA	NA
15	Vu08:32088851..32188851	*Vigun08g148600*	Vu08	32105529	32108916	PFNANANA76	RNA recognition motif. (a.k.a. RRM, RBD, or RNP domain)
15	Vu08:32088851..32188851	*Vigun08g148700*	Vu08	32110363	32113710	PFNANA168	C2 domain
15	Vu08:32088851..32188851	*Vigun08g148800*	Vu08	32116183	32117842	PFNANA168	C2 domain
15	Vu08:32088851..32188851	*Vigun08g148900*	Vu08	32123303	32124711	PFNANA168	C2 domain
15	Vu08:32088851..32188851	*Vigun08g149000*	Vu08	32125703	32128501	PFNA4784	Protein of unknown function, DUF547
15	Vu08:32088851..32188851	*Vigun08g149100*	Vu08	32129967	32142314	PF14796	Clathrin-adaptor complex-3 beta-1 subunit C-terminal
15	Vu08:32088851..32188851	*Vigun08g149200*	Vu08	32145263	32153439	PFNA3468	XS domain
15	Vu08:32088851..32188851	*Vigun08g149300*	Vu08	32154258	32156301	PFNA333NA	Rare lipoprotein A (RlpA)-like double-psi beta-barrel
15	Vu08:32088851..32188851	*Vigun08g149400*	Vu08	32166013	32167593	PFNA1728	FtsJ-like methyltransferase
15	Vu08:32088851..32188851	*Vigun08g149500*	Vu08	32167938	32174751	PFNANANA22	Actin
15	Vu08:32088851..32188851	*Vigun08g149600*	Vu08	32180340	32180837	NA	NA
16	Vu08:32986301..33086301	*Vigun08g157200*	Vu08	32987543	32991774	PFNA2469	Fasciclin domain
16	Vu08:32986301..33086301	*Vigun08g157300*	Vu08	32998113	33001965	NA	NA
16	Vu08:32986301..33086301	*Vigun08g157400*	Vu08	33002902	33003444	NA	NA
16	Vu08:32986301..33086301	*Vigun08g157500*	Vu08	33005657	33005842	NA	NA
16	Vu08:32986301..33086301	*Vigun08g157600*	Vu08	33010241	33014698	PFNA9258	Glycosyl transferase family 64 domain
16	Vu08:32986301..33086301	*Vigun08g157700*	Vu08	33014833	33017386	NA	NA
16	Vu08:32986301..33086301	*Vigun08g157800*	Vu08	33019375	33021196	PFNA7231	Hs1pro-1 N-terminus

16	Vu08:32986301..33086301	*Vigun08g157900*	Vu08	33030606	33032761	PFNANA466	Ribosomal protein L1NA
16	Vu08:32986301..33086301	*Vigun08g158000*	Vu08	33035621	33041839	PFNA5739	SNARE domain
16	Vu08:32986301..33086301	*Vigun08g158100*	Vu08	33056234	33061572	PFNA149NA	Transmembrane amino acid transporter protein
16	Vu08:32986301..33086301	*Vigun08g158200*	Vu08	33071767	33073902	PF1NA44NA	Ubiquitin-binding WIYLD domain
16	Vu08:32986301..33086301	*Vigun08g158300*	Vu08	33074472	33080482	PFNA2163	Peptidase family M5NA
16	Vu08:32986301..33086301	*Vigun08g158400*	Vu08	33083466	33086621	PFNA8263	Leucine rich repeat N-terminal domain
17	Vu08:33023211..33123211	*Vigun08g157900*	Vu08	33030606	33032761	PFNANA466	Ribosomal protein L1NA
17	Vu08:33023211..33123211	*Vigun08g158000*	Vu08	33035621	33041839	PFNA5739	SNARE domain
17	Vu08:33023211..33123211	*Vigun08g158100*	Vu08	33056234	33061572	PFNA149NA	Transmembrane amino acid transporter protein
17	Vu08:33023211..33123211	*Vigun08g158200*	Vu08	33071767	33073902	PF1NA44NA	Ubiquitin-binding WIYLD domain
17	Vu08:33023211..33123211	*Vigun08g158300*	Vu08	33074472	33080482	PFNA2163	Peptidase family M5NA
17	Vu08:33023211..33123211	*Vigun08g158400*	Vu08	33083466	33086621	PFNA8263	Leucine rich repeat N-terminal domain
17	Vu08:33023211..33123211	*Vigun08g158500*	Vu08	33092241	33103289	PFNANA56NA	Leucine Rich Repeat
17	Vu08:33023211..33123211	*Vigun08g158600*	Vu08	33109004	33112450	PFNANA56NA	Leucine Rich Repeat
18	Vu08:33053051..33153051	*Vigun08g158100*	Vu08	33056234	33061572	PFNA149NA	Transmembrane amino acid transporter protein
18	Vu08:33053051..33153051	*Vigun08g158200*	Vu08	33071767	33073902	PF1NA44NA	Ubiquitin-binding WIYLD domain
18	Vu08:33053051..33153051	*Vigun08g158300*	Vu08	33074472	33080482	PFNA2163	Peptidase family M5NA
18	Vu08:33053051..33153051	*Vigun08g158400*	Vu08	33083466	33086621	PFNA8263	Leucine rich repeat N-terminal domain
18	Vu08:33053051..33153051	*Vigun08g158500*	Vu08	33092241	33103289	PFNANA56NA	Leucine Rich Repeat
18	Vu08:33053051..33153051	*Vigun08g158600*	Vu08	33109004	33112450	PFNANA56NA	Leucine Rich Repeat
18	Vu08:33053051..33153051	*Vigun08g158700*	Vu08	33125329	33127752	PFNANA56NA	Leucine Rich Repeat
18	Vu08:33053051..33153051	*Vigun08g158800*	Vu08	33127977	33130982	PFNANA56NA	Leucine Rich Repeat
18	Vu08:33053051..33153051	*Vigun08g158900*	Vu08	33140515	33143964	PFNA8263	Leucine rich repeat N-terminal domain
18	Vu08:33053051..33153051	*Vigun08g159000*	Vu08	33152224	33155475	PFNANANA69	Protein kinase domain
19	Vu08:33214011..33314011	*Vigun08g159400*	Vu08	33218841	33222955	PFNA8263	Leucine rich repeat N-terminal domain
19	Vu08:33214011..33314011	*Vigun08g159500*	Vu08	33226734	33230264	PFNA8263	Leucine rich repeat N-terminal domain
19	Vu08:33214011..33314011	*Vigun08g159600*	Vu08	33235991	33239410	PFNANA56NA	Leucine Rich Repeat
19	Vu08:33214011..33314011	*Vigun08g159700*	Vu08	33241208	33244723	PFNA8263	Leucine rich repeat N-terminal domain
19	Vu08:33214011..33314011	*Vigun08g159800*	Vu08	33244776	33245684	NA	NA
19	Vu08:33214011..33314011	*Vigun08g159900*	Vu08	33248051	33253930	NA	NA
19	Vu08:33214011..33314011	*Vigun08g160000*	Vu08	33254287	33257296	PFNA3226	Yippee zinc-binding/DNA-binding /Mis18, centromere assembly
19	Vu08:33214011..33314011	*Vigun08g160100*	Vu08	33270031	33274713	PFNANANA67	Cytochrome P45NA
19	Vu08:33214011..33314011	*Vigun08g160200*	Vu08	33277414	33280317	PFNANA466	Ribosomal protein L1NA
19	Vu08:33214011..33314011	*Vigun08g160300*	Vu08	33283577	33284646	NA	NA
19	Vu08:33214011..33314011	*Vigun08g160400*	Vu08	33287959	33289210	PFNA7NA11	Protein of unknown function (DUF1313)
19	Vu08:33214011..33314011	*Vigun08g160500*	Vu08	33292050	33295837	PFNA9279	Phosphoinositide-specific phospholipase C, efhand-like
19	Vu08:33214011..33314011	*Vigun08g160600*	Vu08	33295838	33301482	PFNA9279	Phosphoinositide-specific phospholipase C, efhand-like
19	Vu08:33214011..33314011	*Vigun08g160700*	Vu08	33312867	33317990	PFNANA168	C2 domain

20	Vu08:33228221..33328221	*Vigun08g159500*	Vu08	33226734	33230264	PFNA8263	Leucine rich repeat N-terminal domain
20	Vu08:33228221..33328221	*Vigun08g159600*	Vu08	33235991	33239410	PFNANA56NA	Leucine Rich Repeat
20	Vu08:33228221..33328221	*Vigun08g159700*	Vu08	33241208	33244723	PFNA8263	Leucine rich repeat N-terminal domain
20	Vu08:33228221..33328221	*Vigun08g159800*	Vu08	33244776	33245684	NA	NA
20	Vu08:33228221..33328221	*Vigun08g159900*	Vu08	33248051	33253930	NA	NA
20	Vu08:33228221..33328221	*Vigun08g160000*	Vu08	33254287	33257296	PFNA3226	Yippee zinc-binding/DNA-binding /Mis18, centromere assembly
20	Vu08:33228221..33328221	*Vigun08g160100*	Vu08	33270031	33274713	PFNANANA67	Cytochrome P45NA
20	Vu08:33228221..33328221	*Vigun08g160200*	Vu08	33277414	33280317	PFNANA466	Ribosomal protein L1NA
20	Vu08:33228221..33328221	*Vigun08g160300*	Vu08	33283577	33284646	NA	NA
20	Vu08:33228221..33328221	*Vigun08g160400*	Vu08	33287959	33289210	PFNA7NA11	Protein of unknown function (DUF1313)
20	Vu08:33228221..33328221	*Vigun08g160500*	Vu08	33292050	33295837	PFNA9279	Phosphoinositide-specific phospholipase C, efhand-like
20	Vu08:33228221..33328221	*Vigun08g160600*	Vu08	33295838	33301482	PFNA9279	Phosphoinositide-specific phospholipase C, efhand-like
20	Vu08:33228221..33328221	*Vigun08g160700*	Vu08	33312867	33317990	PFNANA168	C2 domain
20	Vu08:33228221..33328221	*Vigun08g160800*	Vu08	33318706	33319571	NA	NA
20	Vu08:33228221..33328221	*Vigun08g160900*	Vu08	33319616	33322826	PF1354NA	Regulator of chromosome condensation (RCC1) repeat
21	Vu08:35985191..36085191	*Vigun08g192500*	Vu08	35977515	35985546	PF1NA392	Golgi transport complex subunit 5
21	Vu08:35985191..36085191	*Vigun08g192600*	Vu08	35985550	35985735	NA	NA
21	Vu08:35985191..36085191	*Vigun08g192700*	Vu08	35988731	35992724	PF1457NA	RING/Ubox like zinc-binding domain
21	Vu08:35985191..36085191	*Vigun08g192800*	Vu08	35993877	36000454	PFNANANA69	Protein kinase domain
21	Vu08:35985191..36085191	*Vigun08g192900*	Vu08	36006420	36009245	PFNA31NA6	WRKY DNA -binding domain
21	Vu08:35985191..36085191	*Vigun08g193000*	Vu08	36014499	36017112	NA	NA
21	Vu08:35985191..36085191	*Vigun08g193100*	Vu08	36021043	36021725	PF13639	Ring finger domain
21	Vu08:35985191..36085191	*Vigun08g193200*	Vu08	36022296	36025866	NA	NA
21	Vu08:35985191..36085191	*Vigun08g193300*	Vu08	36029405	36032775	PFNA7885	Ion channel
21	Vu08:35985191..36085191	*Vigun08g193400*	Vu08	36033452	36034198	PFNA3479	Domain of unknown function (DUF296)
21	Vu08:35985191..36085191	*Vigun08g193500*	Vu08	36034915	36036925	PF12854	PPR repeat
21	Vu08:35985191..36085191	*Vigun08g193600*	Vu08	36037515	36042197	PFNA7983	X8 domain
21	Vu08:35985191..36085191	*Vigun08g193700*	Vu08	36045705	36048145	PFNA1553	Acyltransferase
21	Vu08:35985191..36085191	*Vigun08g193800*	Vu08	36051501	36056724	PFNA1896	Eukaryotic and archaeal DNA primase small subunit
21	Vu08:35985191..36085191	*Vigun08g193900*	Vu08	36060241	36066240	PFNA6293	Lipopolysaccharide kinase (Kdo/WaaP) family
21	Vu08:35985191..36085191	*Vigun08g194000*	Vu08	36068503	36068676	NA	NA
21	Vu08:35985191..36085191	*Vigun08g194100*	Vu08	36068893	36071027	NA	NA
21	Vu08:35985191..36085191	*Vigun08g194200*	Vu08	36074464	36076706	NA	NA
21	Vu08:35985191..36085191	*Vigun08g194300*	Vu08	36077648	36080290	NA	NA
21	Vu08:35985191..36085191	*Vigun08g194400*	Vu08	36083454	36085912	PFNANANA67	Cytochrome P45NA
22	Vu10:33175011..33275011	*Vigun10g123400*	Vu10	33163225	33184325	PFNANA931	NB-ARC domain
22	Vu10:33175011..33275011	*Vigun10g123500*	Vu10	33187695	33194242	PFNA2881	SRP54-type protein, helical bundle domain
22	Vu10:33175011..33275011	*Vigun10g123600*	Vu10	33196448	33200778	PFNA3NA61	Thioesterase superfamily

22	Vu10:33175011..33275011	*Vigun10g123700*	Vu10	33202867	33206431	PFNA3NA61	Thioesterase superfamily
22	Vu10:33175011..33275011	*Vigun10g123800*	Vu10	33215981	33218175	PFNANA168	C2 domain
22	Vu10:33175011..33275011	*Vigun10g123900*	Vu10	33221042	33224857	PFNA137NA	NAD dependent epimerase/dehydratase family
22	Vu10:33175011..33275011	*Vigun10g124000*	Vu10	33225000	33226340	PF11623	Protein of unknown function (DUF3252)
22	Vu10:33175011..33275011	*Vigun10g124100*	Vu10	33226846	33232236	NA	NA
22	Vu10:33175011..33275011	*Vigun10g124200*	Vu10	33236137	33244519	PFNANA271	Helicase conserved C-terminal domain
22	Vu10:33175011..33275011	*Vigun10g124300*	Vu10	33246096	33246479	PFNA2519	Auxin responsive protein
22	Vu10:33175011..33275011	*Vigun10g124400*	Vu10	33256161	33268338	PFNANA271	Helicase conserved C-terminal domain
23	Vu10:33176961..33276961	*Vigun10g123400*	Vu10	33163225	33184325	PFNANA931	NB-ARC domain
23	Vu10:33176961..33276961	*Vigun10g123500*	Vu10	33187695	33194242	PFNA2881	SRP54-type protein, helical bundle domain
23	Vu10:33176961..33276961	*Vigun10g123600*	Vu10	33196448	33200778	PFNA3NA61	Thioesterase superfamily
23	Vu10:33176961..33276961	*Vigun10g123700*	Vu10	33202867	33206431	PFNA3NA61	Thioesterase superfamily
23	Vu10:33176961..33276961	*Vigun10g123800*	Vu10	33215981	33218175	PFNANA168	C2 domain
23	Vu10:33176961..33276961	*Vigun10g123900*	Vu10	33221042	33224857	PFNA137NA	NAD dependent epimerase/dehydratase family
23	Vu10:33176961..33276961	*Vigun10g124000*	Vu10	33225000	33226340	PF11623	Protein of unknown function (DUF3252)
23	Vu10:33176961..33276961	*Vigun10g124100*	Vu10	33226846	33232236	NA	NA
23	Vu10:33176961..33276961	*Vigun10g124200*	Vu10	33236137	33244519	PFNANA271	Helicase conserved C-terminal domain
23	Vu10:33176961..33276961	*Vigun10g124300*	Vu10	33246096	33246479	PFNA2519	Auxin responsive protein
23	Vu10:33176961..33276961	*Vigun10g124400*	Vu10	33256161	33268338	PFNANA271	Helicase conserved C-terminal domain
23	Vu10:33176961..33276961	*Vigun10g124500*	Vu10	33275391	33283289	PFNANA39NA	Malic enzyme, N-terminal domain
24	Vu10:36426061..36526061	*Vigun10g146100*	Vu10	36410829	36427496	PF128NA7	Translation initiation factor eIF3 subunit 135
24	Vu10:36426061..36526061	*Vigun10g146200*	Vu10	36428094	36431339	PFNA3188	Eukaryotic cytochrome b561
24	Vu10:36426061..36526061	*Vigun10g146300*	Vu10	36432823	36434780	PFNA7279	Protein of unknown function (DUF1442)
24	Vu10:36426061..36526061	*Vigun10g146400*	Vu10	36438306	36447117	PFNA2212	Dynamin GTPase effector domain
24	Vu10:36426061..36526061	*Vigun10g146500*	Vu10	36459728	36463000	PFNANA24NA	Ubiquitin family
24	Vu10:36426061..36526061	*Vigun10g146600*	Vu10	36464414	36470342	NA	NA
24	Vu10:36426061..36526061	*Vigun10g146700*	Vu10	36475138	36476813	PFNANANA26	Eukaryotic aspartyl protease
24	Vu10:36426061..36526061	*Vigun10g146800*	Vu10	36485781	36486343	NA	NA
24	Vu10:36426061..36526061	*Vigun10g146900*	Vu10	36487123	36488994	PFNANANA1NA	Helix-loop-helix DNA-binding domain
25	Vu10:36643191..36743191	*Vigun10g147900*	Vu10	36646727	36652697	PFNA4NA61	ORMDL family
25	Vu10:36643191..36743191	*Vigun10g148000*	Vu10	36660723	36664789	PFNANANA69	Protein kinase domain
25	Vu10:36643191..36743191	*Vigun10g148100*	Vu10	36666145	36668161	PFNA3226	Yippee zinc-binding/DNA-binding /Mis18, centromere assembly
25	Vu10:36643191..36743191	*Vigun10g148200*	Vu10	36669436	36670893	PF13812	Pentatricopeptide repeat domain
25	Vu10:36643191..36743191	*Vigun10g148300*	Vu10	36679803	36680954	PF13833	EF-hand domain pair
25	Vu10:36643191..36743191	*Vigun10g148400*	Vu10	36682027	36685127	PFNANANA76	RNA recognition motif. (a.k.a. RRM, RBD, or RNP domain)
25	Vu10:36643191..36743191	*Vigun10g148500*	Vu10	36691120	36697022	PF13246	Putative hydrolase of sodium-potassium ATPase alpha subunit
25	Vu10:36643191..36743191	*Vigun10g148600*	Vu10	36695216	36699630	PFNA1535	PPR repeat
25	Vu10:36643191..36743191	*Vigun10g148700*	Vu10	36700690	36706752	PFNA5659	Arabidopsis broad-spectrum mildew resistance protein RPW8

25	Vu10:36643191..36743191	*Vigun10g148800*	Vu10	36718119	36719993	PFNANANA25	ADP-ribosylation factor family
25	Vu10:36643191..36743191	*Vigun10g148900*	Vu10	36737209	36739280	PFNANANANA4	ATPase family associated with various cellular activities (AAA)
26	Vu11:23822761..23922761	*Vigun11g080600*	Vu11	23835472	23841414	NA	NA
26	Vu11:23822761..23922761	*Vigun11g080700*	Vu11	23874001	23875467	PF11955	Plant organelle RNA recognition domain
26	Vu11:23822761..23922761	*Vigun11g080800*	Vu11	23877303	23883200	NA	NA
26	Vu11:23822761..23922761	*Vigun11g080900*	Vu11	23888561	23890396	NA	NA
26	Vu11:23822761..23922761	*Vigun11g081000*	Vu11	23894729	23895099	NA	NA
26	Vu11:23822761..23922761	*Vigun11g081100*	Vu11	23899106	23899369	NA	NA
27	Vu11:26308441..26408441	*Vigun11g088000*	Vu11	26310480	26311278	NA	NA
27	Vu11:26308441..26408441	*Vigun11g088100*	Vu11	26340211	26344511	PFNA5NA97	Protein of unknown function (DUF688)
27	Vu11:26308441..26408441	*Vigun11g088200*	Vu11	26357259	26368536	PF14381	Ethylene-responsive protein kinase Le-CTR1
27	Vu11:26308441..26408441	*Vigun11g088300*	Vu11	26368775	26370910	PF12937	F-box-like
27	Vu11:26308441..26408441	*Vigun11g088400*	Vu11	26397268	26398061	NA	NA
28	Vu03:14511131..14611131	*Vigun03g143600*	Vu03	14520316	14523580	PFNA4116	Fatty acid hydroxylase superfamily
28	Vu03:14511131..14611131	*Vigun03g143700*	Vu03	14539020	14540990	PFNA3953	Tubulin C-terminal domain
28	Vu03:14511131..14611131	*Vigun03g143800*	Vu03	14545834	14553801	PFNA2585	GlcNAc-PI de-N-acetylase
28	Vu03:14511131..14611131	*Vigun03g143900*	Vu03	14560082	14567537	PFNANA481	Protein phosphatase 2C
28	Vu03:14511131..14611131	*Vigun03g144000*	Vu03	14567788	14574941	PFNANA815	Histidinol dehydrogenase
28	Vu03:14511131..14611131	*Vigun03g144100*	Vu03	14590308	14598445	PF13847	Methyltransferase domain
28	Vu03:14511131..14611131	*Vigun03g144200*	Vu03	14602742	14605903	NA	NA
29	Vu09:15457411..15557411	*Vigun09g098400*	Vu09	15457252	15458697	NA	NA
29	Vu09:15457411..15557411	*Vigun09g098500*	Vu09	15460886	15462208	NA	NA
29	Vu09:15457411..15557411	*Vigun09g098600*	Vu09	15502377	15506685	PFNANA642	Zinc finger C-x8-C-x5-C-x3-H type (and similar)
29	Vu09:15457411..15557411	*Vigun09g098700*	Vu09	15507366	15508308	NA	NA
29	Vu09:15457411..15557411	*Vigun09g098800*	Vu09	15515611	15572518	PF14288	1,3-beta-glucan synthase subunit FKS1, domain-1
30	Vu09:16011581..16111581	*Vigun09g099400*	Vu09	16007579	16012648	PFNANA583	Acetyltransferase (GNAT) family
30	Vu09:16011581..16111581	*Vigun09g099500*	Vu09	16055606	16055944	NA	NA
30	Vu09:16011581..16111581	*Vigun09g099600*	Vu09	16060980	16071250	PF1NA193	Telomere length regulation protein
30	Vu09:16011581..16111581	*Vigun09g099700*	Vu09	16072460	16075012	PFNA29NA2	Ulp1 protease family, C-terminal catalytic domain
31	Vu09:16139361..16239361	*Vigun09g099800*	Vu09	16182376	16182710	NA	NA

*Genes was listed by selective regions between subspecies and chromosomes. Regions 1-27 was identified between ssp. *unguiculata* and ssp. *sesquipedalis,* while regions 28-31 were identified between ssp. *sesquipedalis* and ssp. *cylindrica*.

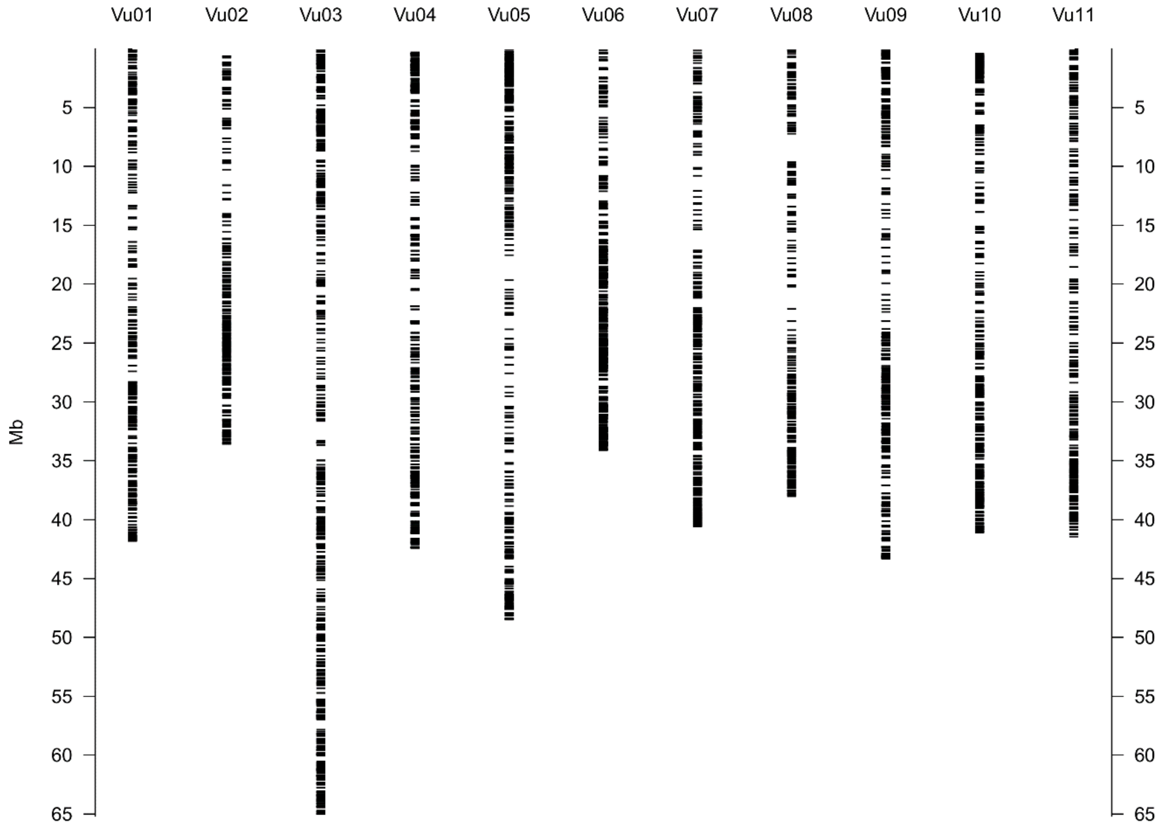

Fig. S1. Distribution of genotyping by sequencing (GBS) identified single nucleotide polymorphism (SNP). Note: Loci found on eleven chromosomes of the *Vigna unguiculata* (IT97K-499-35) cowpea reference genome and scales at the margins of this figure represent sequence length in mega base-pairs.

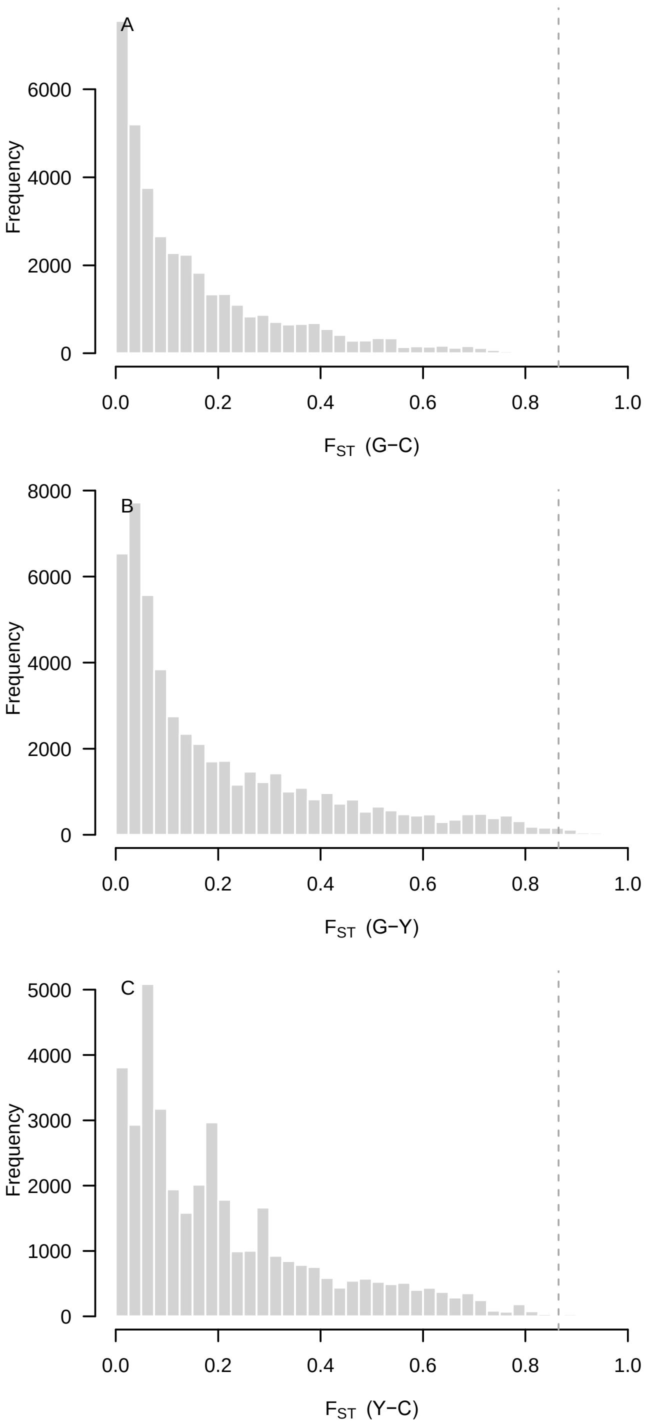

Fig. S2. Frequency distributions of relative differentiation (*F_{ST}*) profiles among three cowpea subspecies. Note: Average pairwise F_{ST} values per sliding window (window size = 100 kb, step size = 10 kb) are shown between: A, grain and fodder cowpea; B, grain and yardlong bean; and C, yardlong and grain cowpea. Dashed vertical lines mark the α = 0.001 threshold for detection of outliers based on whole-genome permutation tests.

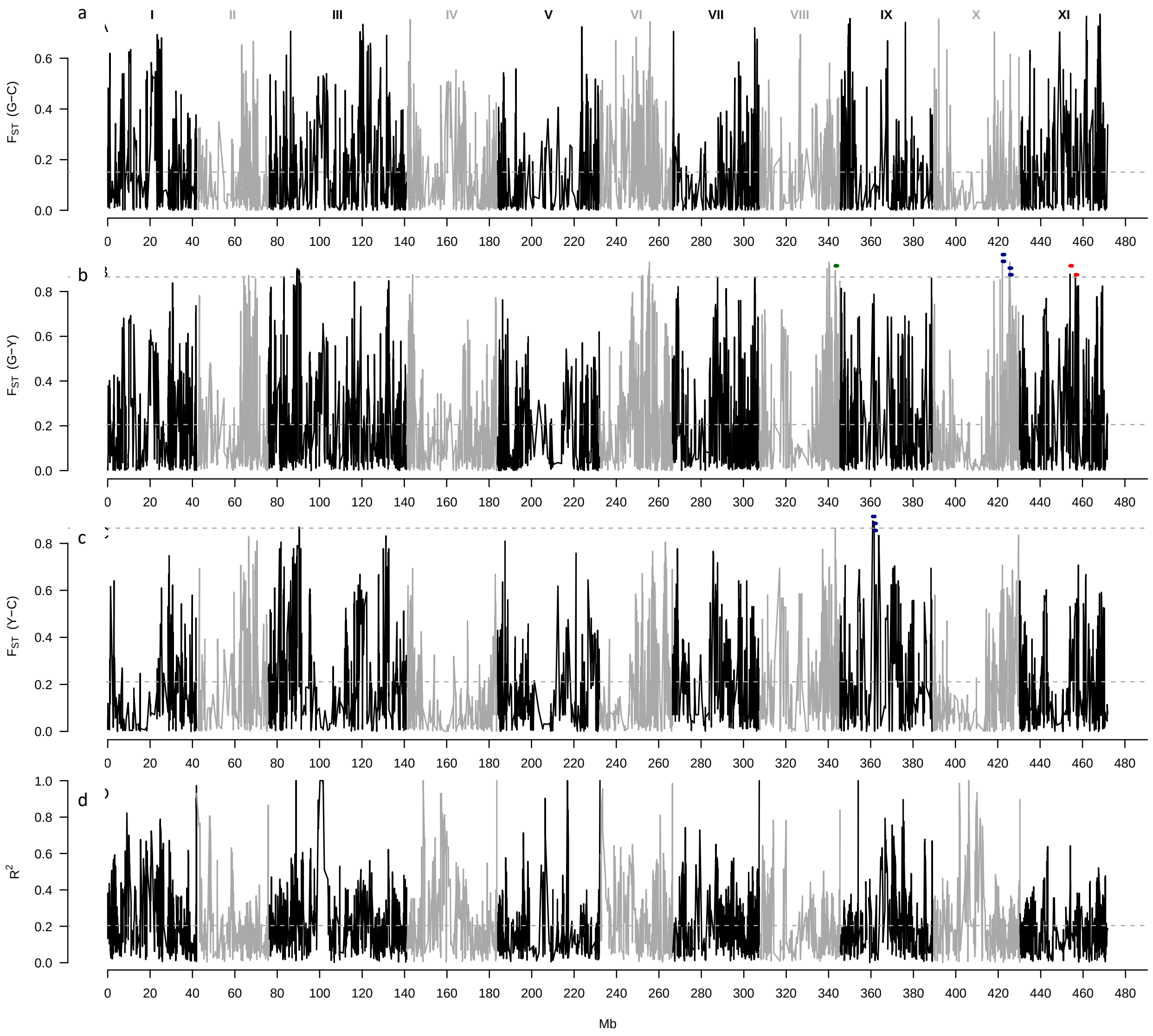

Fig. S3. Genomic landscape of divergence among three cowpea subspecies. Note: Sliding window analyses (window size = 100 kb, step size = 10 kb) are depicted for average relative differentiation (F_{ST}) between: a), grain and fodder cowpea; b) grain and yardlong bean; and c), yardlong and grain cowpea. D), average windowed LD, as measured by R^2, for all accessions. Dashed horizontal lines indicate overall mean and the α = 0.001 threshold (upper line, when applicable, for detection of outliers based on whole-genome permutation tests. Green and blue blocks indicated the outlier regions identified between each comparison. Colored dots above the outlier regions mark known QTLs (Table 2) for pod length (in green), flower scent (in red), and seed coat pattern (in blue). Results of all windowed analyses are plotted against window midpoints in millions of base pairs (Mb). X-axis always shows physical distance in Mb across the 11 chromosomes of cowpea while the Y-axis comparisons always indicate *Fst* value between C (fodder types), G (grain types), and Y (yardlong vegetable types).

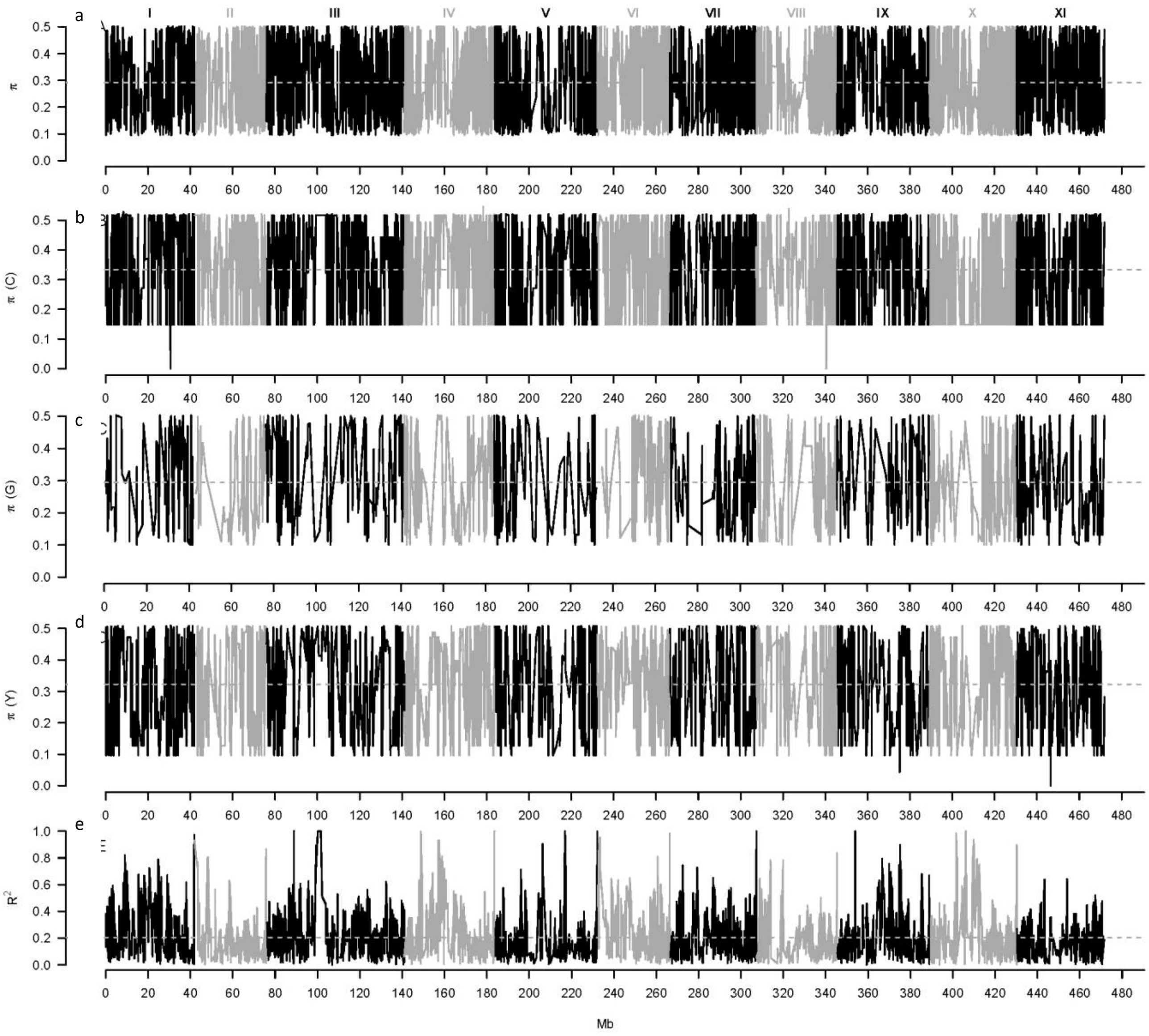

Supplementary Fig. S4. Nucleotide diversity (π) of three cowpea types. a) overall nucleotide diversity; b) fodder types (C); c) grain types (G); d) yardlong vegetable types (Y); e) average windowed LD, as measured by R^2, for all accessions.
